# Orthognathic speech pathology: impacts of Class III malocclusion on speech

**DOI:** 10.1093/ejo/cjab067

**Published:** 2021-09-25

**Authors:** Hillary Lathrop-Marshall, Mary Morgan B Keyser, Samantha Jhingree, Natalie Giduz, Clare Bocklage, Sandrine Couldwell, Haley Edwards, Tim Glesener, Kevin Moss, Sylvia Frazier-Bowers, Ceib Phillips, Timothy Turvey, George Blakey, Ray White, Jeff Mielke, David Zajac, Laura A Jacox

**Affiliations:** Division of Craniofacial and Surgical Care, Orthodontics, Adams School of Dentistry, University of North Carolina, Chapel Hill, USA; Division of Craniofacial and Surgical Care, Orthodontics, Adams School of Dentistry, University of North Carolina, Chapel Hill, USA; Division of Craniofacial and Surgical Care, Orthodontics, Adams School of Dentistry, University of North Carolina, Chapel Hill, USA; Division of Craniofacial and Surgical Care, Orthodontics, Adams School of Dentistry, University of North Carolina, Chapel Hill, USA; Division of Craniofacial and Surgical Care, Orthodontics, Adams School of Dentistry, University of North Carolina, Chapel Hill, USA; Division of Craniofacial and Surgical Care, Oral Surgery, Adams School of Dentistry, University of North Carolina, Chapel Hill, USA; Division of Craniofacial and Surgical Care, Orthodontics, Adams School of Dentistry, University of North Carolina, Chapel Hill, USA; Division of Craniofacial and Surgical Care, Orthodontics, Adams School of Dentistry, University of North Carolina, Chapel Hill, USA; Division of Oral and Craniofacial Health Sciences, Adams School of Dentistry, University of North Carolina, Chapel Hill, USA; Division of Craniofacial and Surgical Care, Orthodontics, Adams School of Dentistry, University of North Carolina, Chapel Hill, USA; Division of Oral and Craniofacial Health Sciences, Adams School of Dentistry, University of North Carolina, Chapel Hill, USA; Division of Craniofacial and Surgical Care, Oral Surgery, Adams School of Dentistry, University of North Carolina, Chapel Hill, USA; Division of Craniofacial and Surgical Care, Oral Surgery, Adams School of Dentistry, University of North Carolina, Chapel Hill, USA; Division of Craniofacial and Surgical Care, Oral Surgery, Adams School of Dentistry, University of North Carolina, Chapel Hill, USA; Department of English, North Carolina State University, Raleigh, USA; Division of Craniofacial and Surgical Care, Craniofacial Center, Adams School of Dentistry, University of North Carolina, Chapel Hill, USA; Division of Craniofacial and Surgical Care, Orthodontics, Adams School of Dentistry, University of North Carolina, Chapel Hill, USA; Division of Oral and Craniofacial Health Sciences, Adams School of Dentistry, University of North Carolina, Chapel Hill, USA

## Abstract

**Introduction:**

Patients with dentofacial disharmonies (DFDs) seek orthodontic care and orthognathic surgery to address issues with mastication, esthetics, and speech. Speech distortions are seen 18 times more frequently in Class III DFD patients than the general population, with unclear causality. We hypothesize there are significant differences in spectral properties of stop (/t/ or /k/), fricative (/s/ or /ʃ/), and affricate (/tʃ/) consonants and that severity of Class III disharmony correlates with the degree of speech abnormality.

**Methods:**

To understand how jaw disharmonies influence speech, orthodontic records and audio recordings were collected from Class III surgical candidates and reference subjects (*n* = 102 Class III, 62 controls). A speech pathologist evaluated subjects and recordings were quantitatively analysed by Spectral Moment Analysis for frequency distortions.

**Results:**

A majority of Class III subjects exhibit speech distortions. A significant increase in the centroid frequency (M1) and spectral spread (M2) was seen in several consonants of Class III subjects compared to controls. Using regression analysis, correlations between Class III skeletal severity (assessed by cephalometric measures) and spectral distortion were found for /t/ and /k/ phones.

**Conclusions:**

Class III DFD patients have a higher prevalence of articulation errors and significant spectral distortions in consonants relative to controls. This is the first demonstration that severity of malocclusion is quantitatively correlated with the degree of speech distortion for consonants, suggesting causation. These findings offer insight into the complex relationship between craniofacial structures and speech distortions.

## Introduction

Speech has influenced human evolution, allowing for knowledge dissemination and advancement of tools (1). However, for patients with speech distortions, there is an implicit assumption that the speaker is of inferior intelligence—this negatively impacts their educational outcomes and quality of life (2). When surveyed, 66 per cent of educators thought communication disorders had an adverse effect on educational development regardless of the child’s intellectual aptitude (3, 4). Pathologic speech impedes communication, impairing social interactions and peer perceptions (5–7). The psychological ramifications of speech sound disorders (SSDs) are also long term and significant. Intellectually normal patients with moderate articulation disorders had reduced career performance relative to unaffected peers, when followed over 28 years (8). Male adult speakers demonstrating frontal lisping were rated lower than non-lispers in their speaking ability, intelligence, education, masculinity, and friendliness (9). Among women adult speakers, lateral lisps drew adverse attention and speakers were judged to be handicapped (10). Articulation errors have a large impact on perception and quality of life.

The orthodontic profession focuses on building healthy smiles that boost patients’ confidence. For the general population, this is geared toward improving esthetics, but for 2.5 per cent of the US population, the discrepancy of the teeth and jaws is handicapping with difficulty masticating, breathing, and speaking (11). Therefore, the role of the orthodontist extends beyond straightening teeth, but includes addressing problems related to jaw function and requires a knowledge of the physiologic interplay of all craniofacial systems.

**Figure 1.**
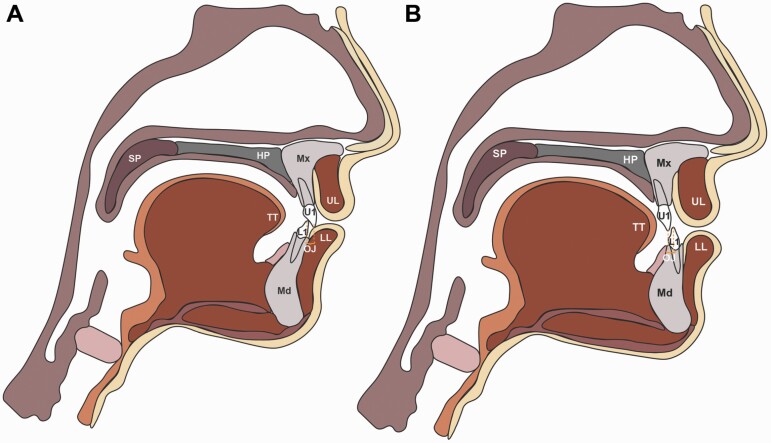
Sagittal schematics of jaw position and craniofacial structures. (A) Schematic of Class I anatomy. (B) Schematic of Class III anatomy with mandibular prognathism and maxillary retrognathism. A Class III skeletal relationship may result from maxillary deficiency, mandibular excess, or a combination of both. One type of Class III jaw relationship is shown here (maxillary deficiency and mandibular excess), though variations exist within Class III malocclusions with differing degrees of upper and lower jaw involvement. Anterior space is commonly reduced in Class III patients, with decreased or negative overjet (OJ) as the maxillary incisors are retruded relative to the mandibular incisors. Class III patients can also present with proclined maxillary incisors, retroclined mandibular incisors, condylar hyperplasia, anterior positioning of the condyle, a short anterior cranial base length, acute cranial base angle, an obtuse gonial angle, and an excessive lower anterior face height (if high angle) (32,33). Class III patients present with a range of severity and combinations of these features. Our large DFD database encompasses a spectrum of Class III presentations, quantified by occlusal and cephalometric measurements (Figure 2B). Labels: U1 = upper 1, L1 = lower 1, UL = upper lip, LL = lower lip, SP = soft palate (or velum), HP = hard palate, TT = tongue tip, Mx = maxilla, and Md = mandible. OJ is the extent of horizontal (anterior–posterior) overlap of the maxillary central incisors over the mandibular central incisors.

Speech formation requires complex coordination of air flow against articulating structures including the tongue, cheeks, teeth, and alveolus (Figure 1). Nearly 90 per cent of English consonants involve articulation in the anterior oral cavity, suggesting occlusal and jaw relationships may affect articulation (12–15). It follows that pathologic speech can occur when the oral cavity is deformed, leading to obligatory and, perhaps, compensatory distortions (16, 17). Patients with dentofacial disharmony (DFD) have severe malocclusions, associated with aberrant jaw function and psychosocial concerns (Figure 1) (18). Among DFD patients, 90 per cent of Class III and 80 per cent of open-bite surgical patients suffer from speech distortions, as compared to 4.9 per cent of adolescents and 3.5 per cent of adults in the general US population (4, 12, 19). The large discrepancy in the incidence of articulation errors suggests a link between jaw disproportion and articulation. Historical studies exploring this topic had smaller sample sizes, were not stratified by vertical and anterior–posterior (AP) classification, and the data were qualitative based on speech pathologists’ assessment of articulation errors (20–24). More recently, quantification of speech quality has appeared in the literature. Smaller studies conducted in non-English languages provide encouraging data on speech quantification; results suggest DFD patients’ speech changes following surgery (25–27). However, a detailed preoperative speech analysis with large patient numbers, stratified by AP and vertical, is currently missing from the literature.

**Figure 2.**
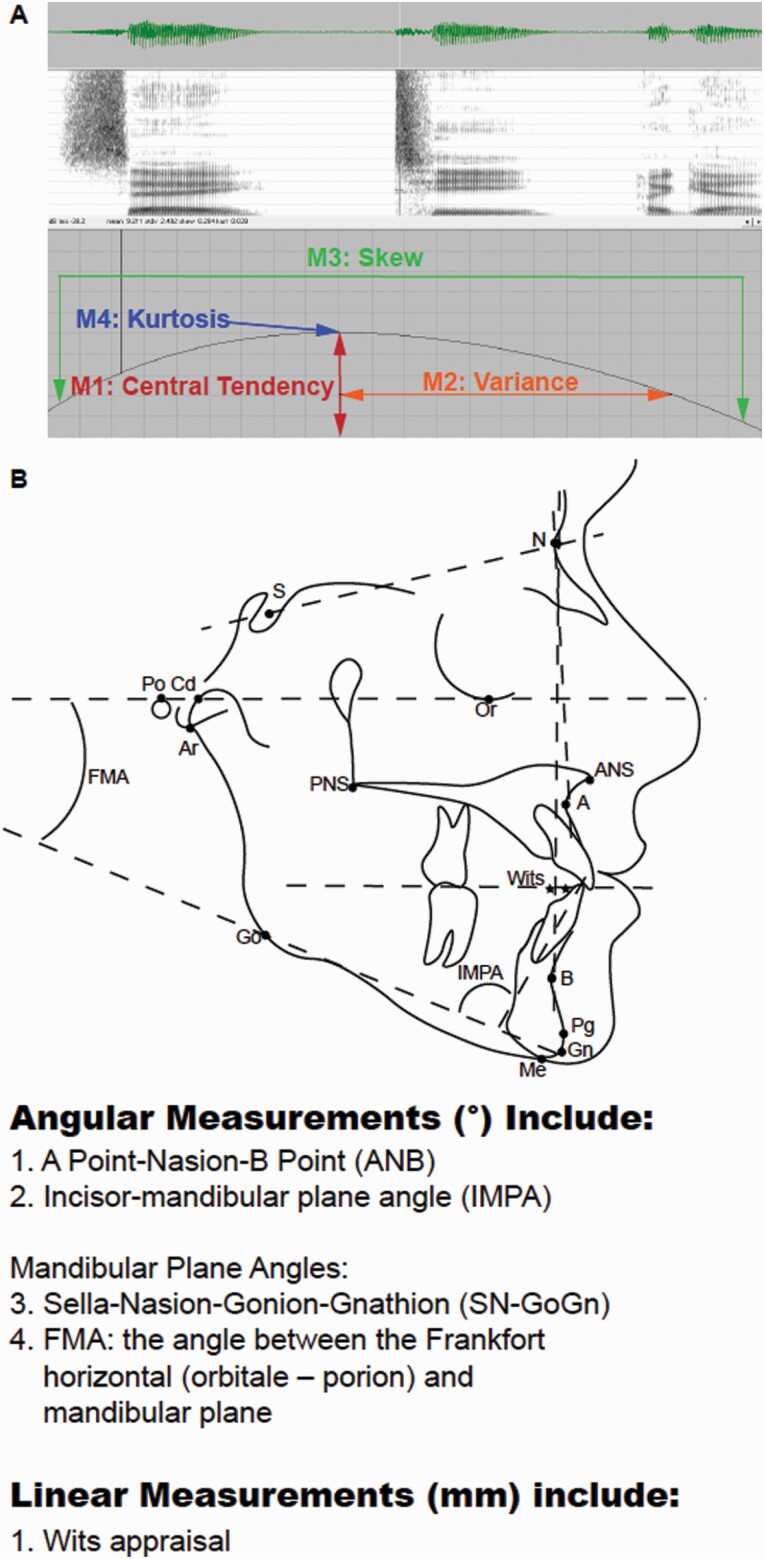
Spectral moment and cephalometric measurements. (A) Spectrogram of a sound wave demonstrating the four spectral moments. The first spectral moment is the central or centroid tendency and is the mean frequency of the sound energy (M1, measured in kHz) (28,29). The second moment (M2, measured in kHz) is the spectral spread (standard deviation or variance) of sound energy; it indicates the spectral spread over which the sound energy is distributed (28,29). The third spectral moment is the tilt or skewness of the sound energy curve (M3, unitless value). And the fourth spectral moment is the kurtosis, or peakedness, of the energy concentration (M4, unitless value). (B) Cephalometric analyses are used to evaluate anterior–posterior positions of the craniofacial skeleton.

One method by which clinician scientists can attain quantitative metrics on sound production is via Spectral Moment Analysis (SMA). This tool relies on statistical descriptors to define characteristics of a sound wave, including the mean or centroid tendency of energy distribution (M1) and the spectral spread of sound energies (M2, Figure 2A) (28, 29). This is a validated method of assessment used in children with cleft lip and palate to understand how palatal morphology impacts speech (30, 31).

The purpose of this study is to use SMA on speech recordings from the DFD population to evaluate how the severity of Class III malocclusion correlates with speech distortion of consonants. Results will allow us to better understand the role of the jaws and teeth in sound generation. We hypothesize there are significant differences in spectral properties of stop (/t/ or /k/), fricative (/s/ or /ʃ/), and affricate (/tʃ/) consonants and that severity of Class III disharmony correlates with the degree of speech abnormality. To test our hypothesis, speech distortions of a Class III population (*n* = 102) and control group (*n* = 62) were perceptually evaluated by a speech pathologist and quantified with SMA. Data were evaluated for linear correlations between the severity of malocclusion, using occlusal and cephalometric measures, and shifts in the spectral moments of five consonants. With the knowledge generated from this investigation, we will provide insight into the physiologic interplay between the jaws, teeth, and vocal instrument and explore how jaw disharmonies are linked to speech disorders.

## Methods

This observational cohort study compared audio, occlusal, and cephalometric data from a control/reference population with DFD patients presenting with severe Class III malocclusions (*n* = 102 Class III, 62 controls). Data were collected in the DFD clinic of the University of North Carolina—Chapel Hill (UNC-CH) Adams School of Dentistry (ASOD), where cases were referred for orthodontic and orthognathic surgical planning (34). UNC is the major referral centre for orthognathic surgery of DFD patients in North Carolina, allowing us to study the relationship between jaw disharmony and speech in a large cohort. Orthodontic and surgical records were collected including occlusal measurements, dental models, photos (intraoral and extraoral), panorex, and cephalogram radiographs. Occlusal measurements captured dental vertical (overbite [OB]), and anteroposterior relationships including overjet (OJ), canine, premolar, and molar dental relationships. This study was conducted in parallel with speech studies of DFD patients with vertical discrepancies (open bites) and Class II malocclusions (35, 36).

Detailed inclusion and exclusion criteria for control participants and DFD patients with Class III malocclusions are summarized in Table 1. Reference subjects were recruited from the UNC ASOD and UNC-CH who had ideal dental and skeletal proportions, including Class I jaw and dental relationships and positive OB (0 mm < OB <4 mm). Class III DFD patients were determined to need orthodontics and orthognathic surgery by board-certified orthodontists and oral surgeons (occlusal criteria in Table 1). DFD patients present either before treatment (no appliances) or mid-treatment with labial appliances. The presence of labial appliances (including brackets, hooks, and/or banded molars) does not affect speech, as long as more than 1 month has elapsed since bonding, for speech adaptation (27, 37–40). Therefore, patients bonded less than 1 month prior to their visit were excluded. Patients in treatment with lingual appliances, clear aligner treatment, and palatal appliances were also excluded (Table 1).

**Table 1.** Inclusion and exclusion criteria. DFD, dentofacial disharmony.

Cohort	Inclusion criteria	Exclusion criteria
Control reference subjects	- Age 14–40 years old with consent - Class I dental and skeletal relationships	- Excess overjet (OJ > or = 4.00 mm) - Deficient overjet (OJ < or = 0.00 mm; edge-to-edge occlusion, reverse overjet or underbite) - Excess overbite (OB > or = 4.00 mm; deep bite) - Negative overbite (OB < or = 0.00 mm; open bite) - English as a second language - Significant regional accent - Bonded with labial appliances less than 1 month prior to visit - Palatal appliance in place (e.g. palatal arch, expander) - Lingual appliances - Clear aligner appliances - Clinical profile is Class II or Class III as assessed by a board-certified orthodontist - Motor speech disorder - Active, symptomatic temporomandibular joint disorder - History of hearing loss - History of cleft lip and/or palate, facial trauma, prior orthognathic surgery, craniosynostosis and syndromic craniofacial conditions - Significant developmental delay - Currently in or history of speech therapy
Patients with Class III DFD	- Age 14–40 years old with consent - Overjet less than or equal to 0 mm - Class III malocclusion - Diagnosed by a board-certified orthodontist and board-certified oral and maxillofacial surgeon as requiring orthodontic care plus orthognathic surgery for correction of malocclusion	- English as a second language - Significant regional accent - Bonded with labial appliances less than 1 month prior to visit - Palatal appliance in place (e.g. palatal arch, expander) - Lingual appliances - Clear aligner appliances - Motor speech disorder - Active, symptomatic temporomandibular joint disorder - History of hearing loss - History of cleft lip and/or palate, facial trauma, prior orthognathic surgery, craniosynostosis, and syndromic craniofacial conditions - Significant developmental delay - Currently in speech therapy

Class III malocclusions may present with either open or closed bites, and these vertical subgroups were analysed separately and also as one large cohort (‘all Class III’). Patient data were stratified by vertical status using OB (open and closed bites) and by AP classification using OJ. OJ was used for AP stratification because molar classification varies with extraction pattern, and anterior tooth position influences speech generation; posterior buccal segment relationships have been implicated in speech distortions to a lesser degree, and only in combination with mandibular prognathism or retrognathism (13, 41). Records were reviewed for OJ, molar, premolar, and canine AP relationships to ensure OJ and A-point-nasion-B-point Angle (ANB°) was consistent with overall AP severity in DFD subjects.

### Speech analyses

Perceptual speech analysis methods were adapted from Vallino and Thomson (21). SMA methods were adapted from Zajac *et al.* (30). Subjects were perceptually evaluated for auditory and visual distortions of the /ta/, /ti/, /la/, /sa/, /si/, and /sɪsi/ sounds by an experienced speech-language pathologist (SLP), to assess whether the sound was normal, interdental, dental, backed, or lateralized (Supplementary Table 1) (21). These syllables were selected to facilitate identification of the various distortions. Patients were then audio recorded while reading a script of words (stimuli = 20 English words each presented on a screen using Microsoft PowerPoint, three times each, randomly presented for a total of 60 words) (Supplementary Table 2). Words were selected to evaluate consonants most affected by jaw disharmony (21, 42). The 20 words (Supplementary Table 2) focus on five consonant phones that target three specific types of sound production: stop sounds (/t/ and /k/), fricatives (/s/, /ʃ/ usually spelled ‘sh’), and the affricate sound (/tʃ/ usually spelled ‘ch’). Each phone was chosen for its articulation of the tongue to either the palate, alveolus, or in the case of /k/—to the velum. In this way, the selected sounds are distinct from one another. For example, the /t/ has a more anterior placement (the alveolus), whereas the /k/ has a posterior placement (the velum) (Figure 1). Similarly, the /s/ has an alveolar articulation, but the /ʃ/ articulates more palataly. Finally, the affricate /tʃ/ lies between a fricative and a stop in its articulation against the palate.

Patients were directed to read a series of 60 phrases comprising the 20 English words and nested within a carrier phrase (‘say ____ again’) to help simulate spontaneous speech. Data were collected in a sound-attenuated booth (Eckoustic Noise Control Products; Eckel Industries of Canada Limited, Morrisburg, Ontario, Canada) using a head-mounted microphone, with recordings collected on a Kay Pentax Computerized Speech Laboratory (CSL Model 4500; Pentax Medical, New Jersey, USA) (30, 31, 34). CSL software was configured to record at a sampling rate of 44.1 kHz with a low-pass filter at 80 per cent of the Nyquist frequency (~18 kHz).

Using TF32 software (CSpeech Software, Milenkovic, 2005, Madison, WI; http://userpages.chorus.net/cspeech/), sound waves were analysed via the Fast Fourier Transform algorithm using a linear frequency scale, simplifying the wave to resemble a statistical distribution curve within a static window of the spectra. Speech waveforms were analysed for stop (/k/, /t/) and affricate (/tʃ/) sounds by placing the cursor at the start of the release burst to capture the burst of the sound energy, which is an important feature of the articulation. Fricative phones (/s/ and /ʃ/) were taken from a sample of the spectrogram’s midpoint, where the sound energy is estimated to be the highest. In short, sound energy data were extracted for the phones using a 20 ms window placed at the temporal midpoint of the two fricatives (/s/, /ʃ/) and at the beginning of the noise burst for the two stops (/t/, /k/) and the affricate (/tʃ/).

The first spectral moment is the central or centroid tendency and is the mean frequency of the sound energy (M1, measured in kHz) within the 20 ms window (Figure 2A) (28, 29). The second moment (M2, measured in kHz) is the spectral spread (standard deviation or variance) of sound energy; it indicates the spectral spread over which the sound energy is distributed (28, 29). The third spectral moment is the tilt or skewness of the sound energy curve (M3, unitless value). Finally, the fourth spectral moment is the kurtosis, or peakedness, of the energy concentration (M4, unitless value). Each consonant (/k/, /t/, /tʃ/, /s/, /ʃ/) has four words associated with it (e.g. chap, cheap, chew, and chop for /tʃ/ ‘ch’), with each word repeated three times, yielding 12 utterances of the consonant sound (Supplementary Table 2). The spectral moments for a phone are calculated as an average taken from the 12 utterances.

### Statistics

Statistical software (SAS software version 9.4; SAS Institute, Inc., Cary, North Carolina, USA) was used to compare spectral moments within and across groups. For statistical analysis of spectral moments, we utilized a mixed model with the word as the random variable. For SMA, the four spectral moments for each of the five sounds were evaluated for ‘all Class III’ subjects. Then the ‘all Class III’ group was stratified by vertical classification (open versus closed bite) to remove potential confounding effects of vertical discrepancies. The ‘all Class III’, open bite and closed bite Class III cohorts were analysed as unadjusted data, as well as after adjustments for race, age, and sex. Linear regression models were used to evaluate the variation of spectral moments relative to occlusal and skeletal cephalometric measurements (Occlusal values: OJ, Cephalometric values: ANB°, Incisor Mandibular Plane Angle [IMPA°], Wits appraisal, Frankfort-Mandibular Plane Angle [FMA°], and Sella-nasion-gonion-gnathion [SN-GoGn]) (Figure 2B). Potential covariates were included in the multivariable models based upon a significant bivariate association with the outcome (*P* < 0.95). Potential covariates not significantly associated with the outcome pairwise but with borderline significance (0.05 ≤ *P* < 0.02) were added to the adjusted models to create fully adjusted models. These variables were retained in the fully adjusted models based upon either being a significant main effect in the model (*P* < 0.05) or confounding the association between our exposure and outcome by 5 per cent or more. Data are presented as both unadjusted and adjusted for race, age, and sex. Statistical significance was accepted at *P* < 0.05 following Tukey adjustment and was accepted at *P* < 0.01 following Tukey and Bonferroni multiple testing adjustments.

To measure intra-examiner reliability of cephalometric tracing, the concordance correlation coefficient was used (Supplementary Table 3). Cephalogram radiographs were traced by a single examiner, blinded to patient identity, DFD, and speech diagnoses, to evaluate skeletal relationships (Figure 2B). Two weeks later, the same examiner retraced one-quarter of the cephalograms (*n* = 25), randomly chosen, to calculate an intra-examiner concordance correlation (Supplementary Table 3).

Ethics approval was granted by the Institutional Review Board of UNC ASOD (#18-1406 & #19-1196).

## Results

### Subject sample

One hundred and two DFD patients with Class III malocclusions were consecutively enrolled from our DFD clinic, with screening for inclusion and exclusion criteria (Table 1). Sixty-two reference controls with Class I occlusion and skeletal base were recruited from UNC ASOD and UNC-CH (Table 2). Sex distribution was closely split among Class III DFD patients (52.0 per cent female [*n* = 53]; 48.0 per cent male [*n* = 49]). Forrest *et al.* found spectral trends were 90 per cent accurate between men and women; therefore, sex does not significantly influence spectral analysis, though we adjusted for sex to account for the 10 per cent difference in our adjusted values (Figure 5) (28, 29). The DFD cohort was slightly younger than the reference group (DFD: 20.5 younger; Control: 24.4 younger), prompting us to adjust for age, though within the bounded range (14–40 years old), age is unlikely to affect speech, as it develops and matures by 8 years of age (13, 16, 43, 44). There was a greater representation of African Americans in the surgical cohort, prompting us to adjust for race. Surgical subjects had full orthodontic and surgical records collected, with cephalometric tracing to quantify underlying skeletal relationships.

**Table 2.** Sample demographics table. DFD, dentofacial disharmony; OJ, overjet; OB, overbite; ANB, A-point-nasion-B-point; IMPA, incisor mandibular plane; FMA, Frankfort-mandibular plane; SN-GoGn, Sella-nasion-gonion-gnathion.

	Patients with Class III DFD	Control/reference subjects
Age in years (mean)	20.50 (*n* = 102)	24.40 (*n* = 62)
Age in years (range)	12–55	17–38
Gender	52.00% female (*n* = 53) 48.00% male (*n* = 49)	59.70% female (*n* = 37) 40.30% male (*n* = 25)
Race and ethnicity	33.30% African American (*n* = 34) 4.90% Asian/Pacific Islander (*n* = 5) 46.10% Caucasian (*n* = 47) 9.80% Hispanic/Latino (*n* = 10) 5.90% Southeast Asian/Indian (*n* = 6)	9.70% African American (*n* = 6) 8.10% Asian/Pacific Islander (*n* = 5) 66.10% Caucasian (*n* = 41) 8.10% Hispanic/Latino (*n* = 5) 8.10% Southeast Asian/Indian (*n* = 5)
Bonded labial appliances (current braces)	31.40% (*n* = 32)	4.83% (*n* = 3)
OJ mean, range (in mm)	−3.50 (range −17 to 7)	2.44 (range 1 to 4)
OB mean, range (in mm)	0.54 (range −7 to 6)	2.45 (range 1 to 4)
*Ceph mean, ranges:*		
ANB mean, range (in degrees)	−3.07 (range −12.40 to 3.90)	N/A
IMPA (in degrees)	84.95 (range 62.80 to 103.60)	N/A
Wits (mm)	−10 (range −25.20 to 2.50)	N/A
FMA (in degrees)	24.65 (range 10 to 64.6)	N/A
SN-GoGn (in degrees)	30.26 (range 14.10 to 47.60)	N/A

### Perceptual results

**Figure 3.**
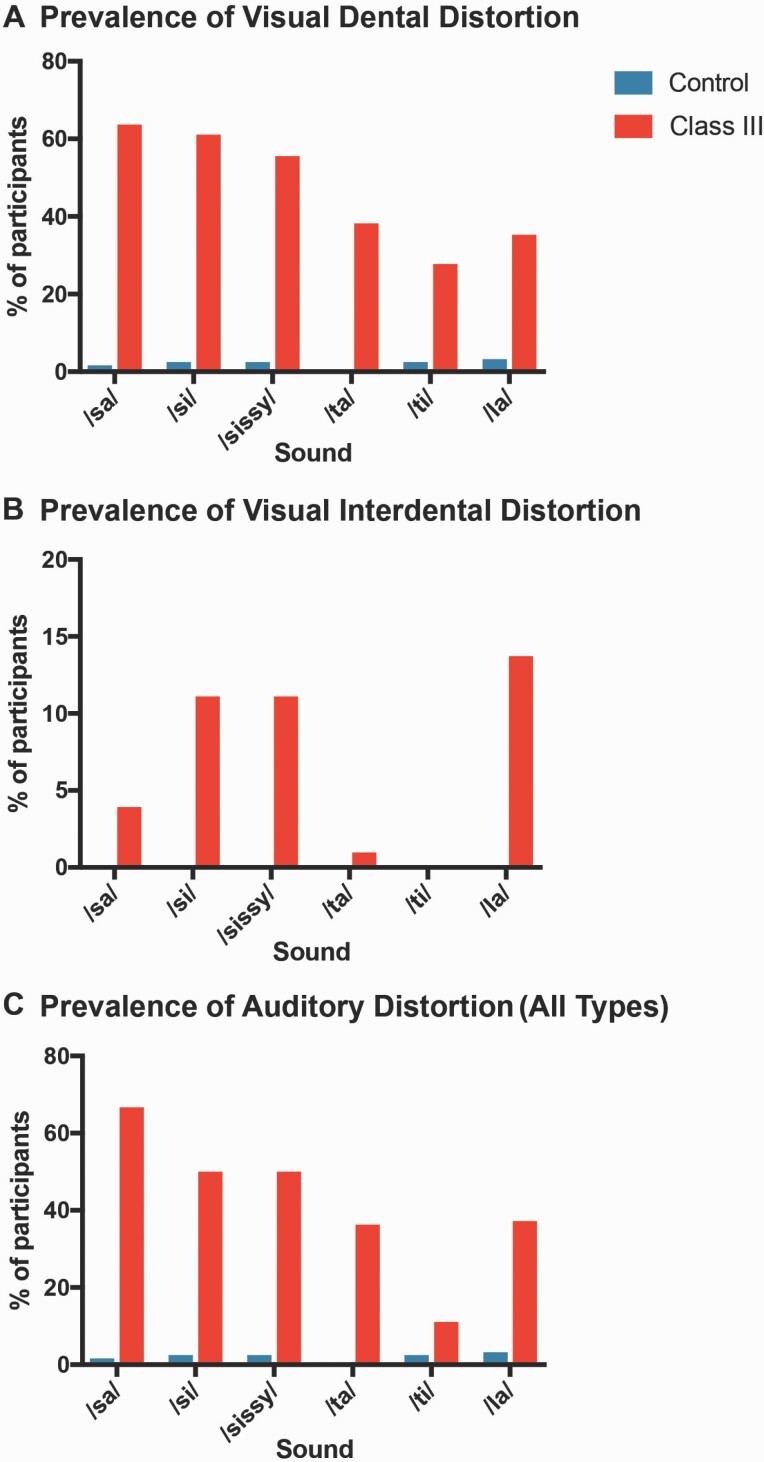
Prevalence of speech distortion in DFD patients with Class III and Class I Controls. A speech-language pathologist performed a perceptual evaluation and scored patients for visual and auditory distortions. Percentages of participants exhibiting distortions are represented in the bar graphs. (A) Prevalence of visual dental distortion. (B) Prevalence of visual interdental distortion. (C) Prevalence of auditory distortion (any type). An auditory distortion is when a sound or phone is distorted or changed. Types of auditory distortions include whistled, backed, and lateralized (21). Blue: Control patients. Red: DFD patients with Class III malocclusions. Data are represented in Supplementary Table 1.

**Figure 4.**
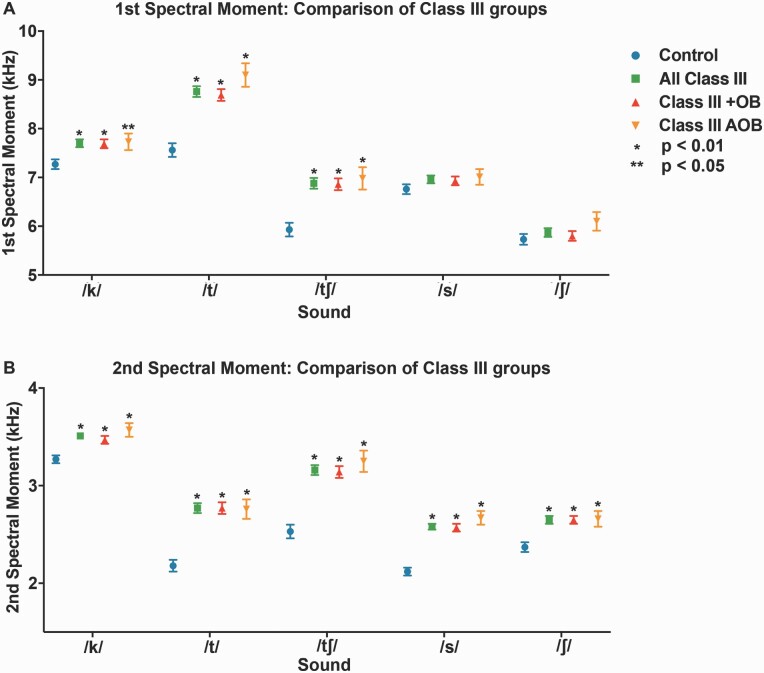
First and second spectral moments for patients with Class III DFD and Class I controls. (A) First spectral moment/mean frequency (M1) by a consonant. (B) Second spectral moment (M2, standard deviation = spectral spread) by consonant. Blue circle: Control. Green square: All Class III DFD patients. RRed triangle: Class III DFD patients with positive overbite (+OB). Orange upside-down triangle: Class III DFD patients with an anterior open bite (AOB) or negative overbite. Bars represent standard deviation. Conventions: *P < 0.01 significant by Bonferroni adjustment. **P < 0.05. Data are represented in Supplementary Tables 4–13.

**Figure 5.**
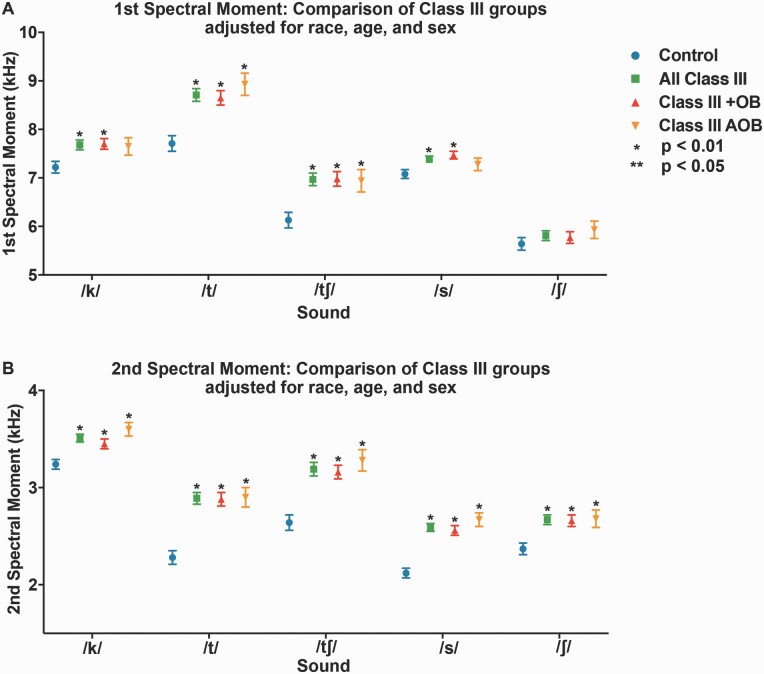
First and second spectral moments for patients with Class III DFD and Class I controls, adjusted for race, sex, and age. (A) First spectral moment/mean frequency (M1) by a consonant. (B) Second spectral moment (M2, standard deviation = spectral spread) by consonant. Blue circle: Control. Green square: All Class III DFD patients. Red triangle: Class III DFD patients with positive overbite (+OB). Orange upside-down triangle: Class III DFD patients with an anterior open bite (AOB) or negative overbite. Bars represent standard error. Conventions: *P < 0.01 significant by Bonferroni adjustment. **P < 0.05. Data are represented in Supplementary Tables 4–13.

SLP perceptual evaluation identified marked differences in the prevalence of speech distortions between DFD subjects and controls. The majority of DFD patients with Class III malocclusion had speech distortions, consistent with existing literature (Figure 3, Supplementary Table 1) (13, 16, 21). The percentage of Class III DFD patients with auditory, visual dental, and interdental distortions of /sa/, /si/, /sɪsi/, /ta/, /ti/, and /la/ was multiple times the prevalence among controls (Figure 3, Supplementary Table 1). An auditory distortion is when a sound or phone is distorted or changed. Types of auditory distortions include whistled, backed, and lateralized (21). A visual distortion occurs when the tongue is positioned abnormally during articulation, with the tongue either pushed against the anterior teeth (dental, dentalized error) or extending through the front teeth (interdental, inderdentalized error) (21). Interdental visual distortions of /sa/, /si/, /sɪsi/, and /la/ were much more prevalent in DFD subjects than controls, but were still seen in less than a fifth of Class III subjects (Figure 3B). However, visual dental and auditory distortions were seen in the majority of Class III subjects, compared to a small minority of controls (<2 per cent) (Figure 3A and C). Notably, Class III patients displayed dentalized distortions for /sa/ sounds, approximately 60 times more frequently than reference subjects (Figure 3, Supplementary Table 1). Consistent with this, quantitative SMA identified significant differences in the first, second, and fourth moments for the /s/ and /t/ sounds when comparing Class III cohorts with reference subjects (Figures 4 and 5, Supplementary Figures 1 and 2). High concordance correlation coefficients indicate repeatable and uniform landmark identification and tracing (Supplementary Table 3).

### Differences in spectral moments between Class III cohorts

Sounds /k/, /t/, and /tʃ/ exhibited significant differences in the first spectral moment (M1 = centroid tendency), between Class III cohorts and controls, when not statistically adjusted for covariates, with the addition of sound /s/ when adjusted (Figures 4 and 5, Supplementary Tables 4–13). All measured sounds (/k/, /t/, /tʃ/, /s/, and /ʃ/) exhibited significant differences from controls in the second spectral moment (M2 = spectral spread) with and without statistical adjustment for covariates (Figures 4 and 5, Supplementary Tables 4–13). Covariates included race, age, and gender. Class III DFD subjects as a group (‘all Class III’) were stratified by OB (‘Class III with OB [+OB]’ and ‘Class III Anterior Open Bite [AOB]’). The ‘Class III + OB’ group produced /k/, /t/, and /tʃ/ consonants with significantly higher mean frequencies (M1s) than controls when unadjusted for covariates, with the addition of consonant /s/ when adjusted. The ‘Class III AOB’ group produced /t/ and /tʃ/ consonants with significantly higher mean frequencies (M1s) than controls, both with and without adjustment for covariates. Both ‘Class III +OB’ and ‘Class III AOB’ groups had significantly higher spectral spread (M2) values across all measured consonants, both with and without adjustment. All M1 and M2 experimental values for the Class III groups and subgroups trended higher than that of controls (Figures 4 and 5, Supplementary Tables 4–13). Patients in the ‘Class III AOB’ subgroup had the greatest differences in these moments, possibly related to the combination of vertical and AP discrepancies.

For the third spectral moment (M3 = skew), there were significant differences between Class III cohorts and reference subjects for /t/ and /tʃ/ sounds with and without covariate adjustments, with a lower positive skew for Class III cohorts (Supplementary Figures 1 and 2, Supplementary Tables 4–13). The fourth spectral moment (M4 = Kurtosis) for sounds /t/, /tʃ/, and /s/ was significantly lower, with less peaked data, for Class III cohorts compared to controls, with and without covariate adjustments (Supplementary Figures 1 and 2, Supplementary Tables 4–13). Modest changes between unadjusted and adjusted data were noted for the /tʃ/ phone in particular subgroups, indicating that age, race, and sex may influence this sound. No significant differences were found between the Class III cohorts and controls for M3 or M4 for /k/ and /ʃ/ sounds, with and without covariate adjustments (Supplementary Figures 1 and 2, Supplementary Tables 4–13).

### Spectral distortion of consonants varies linearly with malocclusion severity

**Figure 6.**
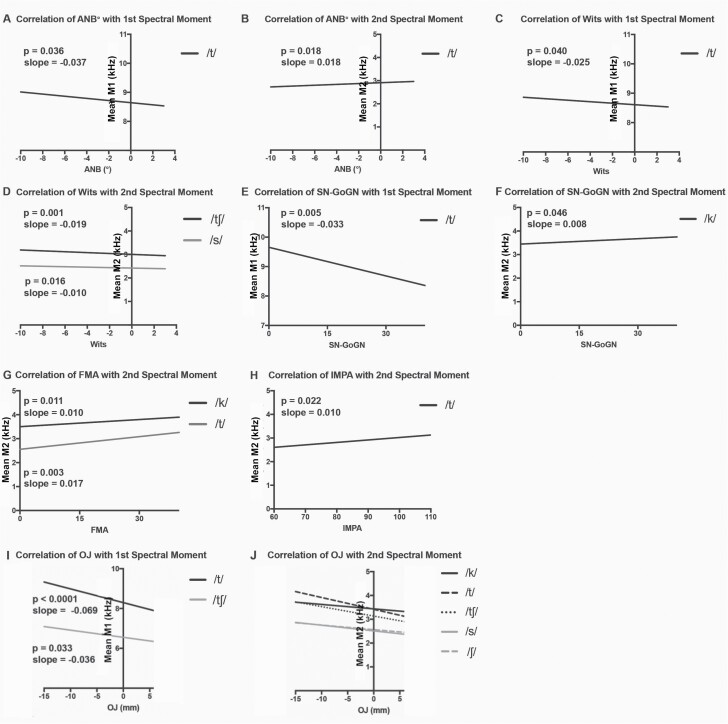
Regression plots of significant linear trends for patients with DFD and Class I controls, adjusted for race, sex, and age. Spectral moments from DFD patients and controls plotted as a function of cephalometric (Class II, III and AOB patient data) and occlusal measures (Class III only), for statistically significant relationships (*P* < 0.05). (A) Correlation of ANB with the first spectral moment (M1). (B) Correlation of ANB with the second spectral moment (M2). (C) Correlation of Wits with M1. (D) Correlation of Wits with M2. (E) Correlation of SN-GoGn with M1. (F) Correlation of Sn-Go-Gn with M2. (G) Correlation of FMA with M2. (H) Correlation of IMPA with M2. (I) Correlation of OJ with M1. (J) Correlation of OJ with M2. *P* values and linear slopes are specified on the graphs for A–I. For J, consonant values follow (*P* value, slope): /k/ (0.0072, −0.01877), /t/ (0.0377, −0.01823), /ch/ (<0.0001, −0.02387), /s/ (0.0001, −0.02387), /sh/ (0.0049, −0.01927). Data are represented in Supplementary Tables 14–19.

Using regression modeling, several linear relationships were found between cephalometric measures and sound spectral moments (Figure 6, Supplementary Tables 14–19). All patient data were included in the model, to represent the full range of AP phenotypes (*n* = 102 Class III, 37 Class II, 39 AOB, 62 controls). Cephalometric measures were evaluated as a reflection of skeletal disproportion and included ANB°, Wits, IMPA°, FMA°, and Sn-GoGn°. For the /t/ phone, the first spectral moment (M1) varies linearly with ANB° and Wits appraisal; as ANB° and Wits appraisal increase in value (indicating a reduction in Class III tendency) the average frequency of M1 decreases (Figure 6A and C). As SN-GoGn° increases (indicating an increase in mandibular plane angle), the average frequency of M1 for /t/ decreases (Figure 6E). No significant trends were noted for M1 as a function of IMPA°, suggesting lower incisor angulation has limited impact on speech; however, M2 increases slightly with increasing IMPA° and ANB° angles for /t/ (Figure 6B and H). M2 decreases modestly with a rise in Wits for the /s/ and /tʃ/ sounds (Figure 6D). The second spectral moment (M2) is also impacted by mandibular plane angle; increases in FMA° and SN-GoGn° linearly correlate with increases in the spectral spread (M2) of the /k/ and /t/ phones (Figure 6F and G).

M1 and M2 of the /t/ phone have significant associations with cephalometric measures, suggesting that articulatory distortions of /t/ are linearly related to the degree of skeletal malocclusion, both in the AP (ANB° and Wits) and vertical (FMA°, SN-GoGn°) dimensions (Figure 6, Supplementary Tables 14–19) (35). The other consonant sounds vary with horizontal and vertical discrepancies, but not linearly, as assessed in this model (Figures 4 and 5, Supplementary Figures 1 and 2).

To evaluate the influence of AP jaw position, linear modeling was conducted for the first spectral moment of each consonant as a function of OJ, with the Class III DFD and control cohorts. Significant relationships exist for /t/ and /tʃ/ where increasing OJ was associated with a decrease in the first spectral moment (Figure 6I, Supplementary Table 19). All five evaluated consonants exhibited a significant inverse linear relationship between M2 and OJ, where spectral spread (M2) decreased as OJ became more positive (less Class III) (Figure 6J, Supplementary Table 19). Linear associations between the first spectral moment (M1) and degree of negative OJ for /t/ and /tʃ/ sounds are consistent with the hypothesis that severity of AP jaw disproportion tracks with the severity of SSD.

## Discussion

In our study, perceptual and quantitative data are consistent with published reports demonstrating higher frequencies of SSD in patients with profound malocclusions (Figure 3) (13, 16, 21). As in the literature, auditory and visual dental distortions were seen in more than half of our Class III DFD subjects and in a small minority of Class I controls (<2 per cent) (Figure 3, Supplementary Table 1). Interdental distortions were also more prevalent in DFD subjects than controls, but were observed in a minority of Class III patients (<20 per cent). Dentalized distortions are more common than interdentalized distortions in our Class III patients. Class III patients appear to have a more anterior constriction location for speech, possibly related to their maxillary deficiency.

Perceptual evaluations, particularly of visual distortions, allow us to evaluate whether Class III patients are presenting with obligatory or compensatory speech distortions. With obligatory errors, articulators including the tongue are positioned properly but the teeth, jaws, and oral anatomy are incorrectly placed causing distortions that require surgical and orthodontic management for correction (17, 18). Compensatory errors occur when abnormal anatomy leads patients to alter articulators to compensate; management requires speech therapy in addition to surgical and orthodontic care for speech correction. A majority of our Class III DFD patients present with visual and auditory distortions, suggesting that speech pathology is a critical part of DFD patient management, especially following surgery and orthodontics (Figure 3, Supplementary Table 1)

The study extends existing perceptual research into the quantitative realm with a large DFD patient cohort. Sufficient patient numbers allowed for stratification by AP and vertical to conduct subgroup comparisons: a cohort analysis missing from the literature. Existing quantitative studies present promising data focussed on vowels with smaller patient populations, without AP and vertical stratification (25–27). This is the first study to utilize SMA to quantitatively evaluate consonants in the DFD population as a function of malocclusion severity and relative to Class I controls. SMA is advantageous as we can use it to rapidly evaluate large patient datasets. Analysis revealed significant mean differences in the spectral moments of five consonants (/k/, /t/, /ʃ/, /s/, and /tʃ/) between patients with Class III DFD and references subjects, with and without statistical adjustments for sex, age, and race (Figures 4 and 5). It is of note that /k/, a posterior velar sound, had an elevated first spectral moment compared to the controls. An important factor in the mean frequency of /k/ is the size of the front cavity, which is related to constriction location. A smaller cavity will increase the frequency of the first spectral moment. In Class III patients, their front cavity is reduced due to maxillary deficiency where the maxilla is too short in the AP (and often transverse) dimensions, reducing the resonating cavity size. This is one likely explanation for the increased M1 for /k/ of Class III DFD patients.

When stratified by OB, the Class III AOB patients had larger increases in spectral moments than Class III subjects with positive OB (closed bites), possibly due to combined vertical and AP discrepancies. Significant differences in spectral moments across consonants in all Class III cohorts relative to controls suggest that AP discrepancies influence speech.

Significant linear relationships were found between spectral moments and measures of Class III malocclusion severity, suggesting that degree of speech abnormality correlates with the extent of disharmony, consistent with our hypothesis (Figure 6, Supplementary Tables 14–19). This was especially true of the /t/ phone where M1 and M2 have significant associations with the degree of malocclusion in the AP (ANB° and Wits) and vertical (FMA°, SN-GoGn°) dimensions (Figure 6) (35). An inverse relationship existed between OJ and M1 of /t/ and /tʃ/, where more negative OJ was associated with higher M1. In contrast, among Class II patients, there is a positive linear relationship between OJ and M1 for /t/ and /tʃ/, where a more positive OJ was associated with higher M1 (Supplementary Figures 3 and 4) (36). For both Class II and III cohorts, M1 of /t/ and /tʃ/ increases with occlusal severity, with controls having the lowest mean M1 frequency. To better understand these trends for future inquiry, we are evaluating the sounds’ spectral shapes and recordings using an analysis of multitaper spectra. Additionally, the Class III cohort had greater M1 spectral differences, smaller M2 spectral spread, and a higher frequency of perceptual distortions than Class II patients (Supplementary Figures 3 and 4) (36). The inability of Class III patients to posture into a Class I position likely contributes to their more severe presentation relative to Class II patients, who can freely posture their mandible into a Class I or milder Class II position (36). Having negative OJ (‘an underbite’) could influence articulation, with the tongue being positioned forward relative to the maxilla, as the maxilla is positioned farther back due to deficiency; this may make it harder for patients to produce consonants, especially stops like /t/ and affricates like /tʃ/, with proper tongue positioning relative to the maxillary alveolus, palate, and teeth. Our ongoing lingual imaging of DFD patients during speech will help to clarify the mechanism underlying these speech distortions.

Our control sample was not perfectly matched to our DFD patients, due to differences in age, racial, and sex distributions (Table 2) Though sex and age variation are unlikely to impact speech analyses, statistical adjustment of these covariates was performed to err on the side of caution (13). Only minor differences were found with and without adjustments, suggesting these covariates had limited effect (Figures 4 and 5, Supplementary Figures 1–4). There was also a difference in the fraction of patients and controls that had fixed buccal appliances. To adjust for this potential confounder, all participants with braces were required to have been bonded more than 1 month prior to data collection. It takes patients 2–3 weeks to adjust to labial appliances, and by 1-month post-bonding, there is no impact on speech articulation with buccal appliances (37–40). However, patients with palatal or lingual appliances or clear aligners were excluded to avoid confounding.

Results from our analyses may lend insight to speech distortions observed with complex craniofacial abnormalities that include Class III malocclusion, such as craniosynostosis and craniofacial clefting after repair (17, 18, 32). This study of Class III DFD patients, without confounding developmental conditions, may help specialists determine relative impacts of craniofacial anomalies on speech in more complex cases.

Data presented are from a single time point pre-operation. Evaluating how speech changes post-operatively, once jaws and teeth are corrected to a Class I anatomy, will yield valuable insight to tease apart the influence of anatomy and muscular habits on speech and allow us to evaluate if surgically correcting jaw disharmonies improves speech. This longitudinal investigation is ongoing, as our patient cohorts are progressing through orthognathic surgery and being recorded at their postoperative visits. Results could help guide the standard of care for patients with DFD and other craniofacial conditions by oral surgeons, orthodontists, and speech therapists (17, 18).

## Conclusions

1. There is a multiple-fold higher prevalence of auditory and visual distortions in patients with Class III DFD when compared to controls.
2. There are statistically significant differences in consonant spectral moments between the Class III DFD population and controls.
3. Degree of spectral moment distortion for certain consonants correlates linearly with the degree of DFD.

## Supplementary Material

cjab067_suppl_Supplementary_Material

## Data Availability

The data that support the findings are included in the supplementary materials. Any further information is available from the corresponding author, upon reasonable request.
