# Supplementary material for: Orthognathic speech pathology: impacts of Class III malocclusion on speech
Source: Eur J Orthod. 2021 Sep 25;44(3):340–51. doi: 10.1093/ejo/cjab067 (PMC9127721; doi:10.1093/ejo/cjab067)
Supplement: cjab067_suppl_Supplementary_Material [file cjab067_suppl_supplementary_material.pdf]

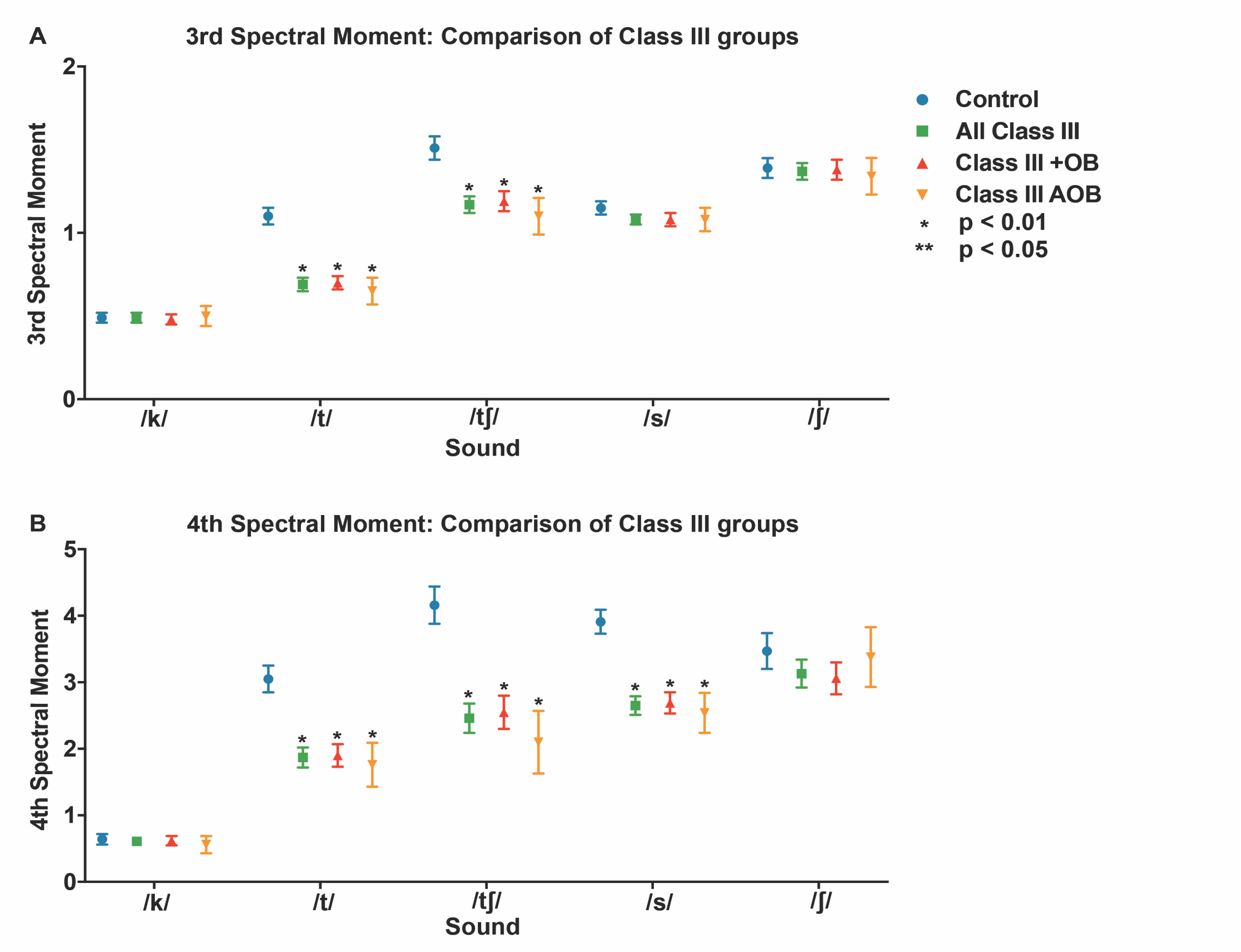

Supplemental Figure 1: 3rd and 4th Spectral Moments for patients with Class III DFD and Class I controls. [A.] 3rd spectral moment (M3, skew = symmetry or degree of distortion of curve) by phone. [B.] 4th spectral moment (M4, kurtosis = tails distribution, measure of outliers). Blue circle: Control. Green square: All Class III DFD patients. Red triangle: Class III DFD patients with positive (+) overbite. Orange upside down triangle: Class III DFD patients with an anterior open bite (AOB) or negative overbite. Bars represent standard deviation. Conventions: * p< 0.01 significant by Bonferonni adjustment. ** p<0.05. ^individual comparison has significance but no type III group significance (p>0.05). ^^ type III significance 0.01<p<0.05. Data are represented in Tables S4-S13.

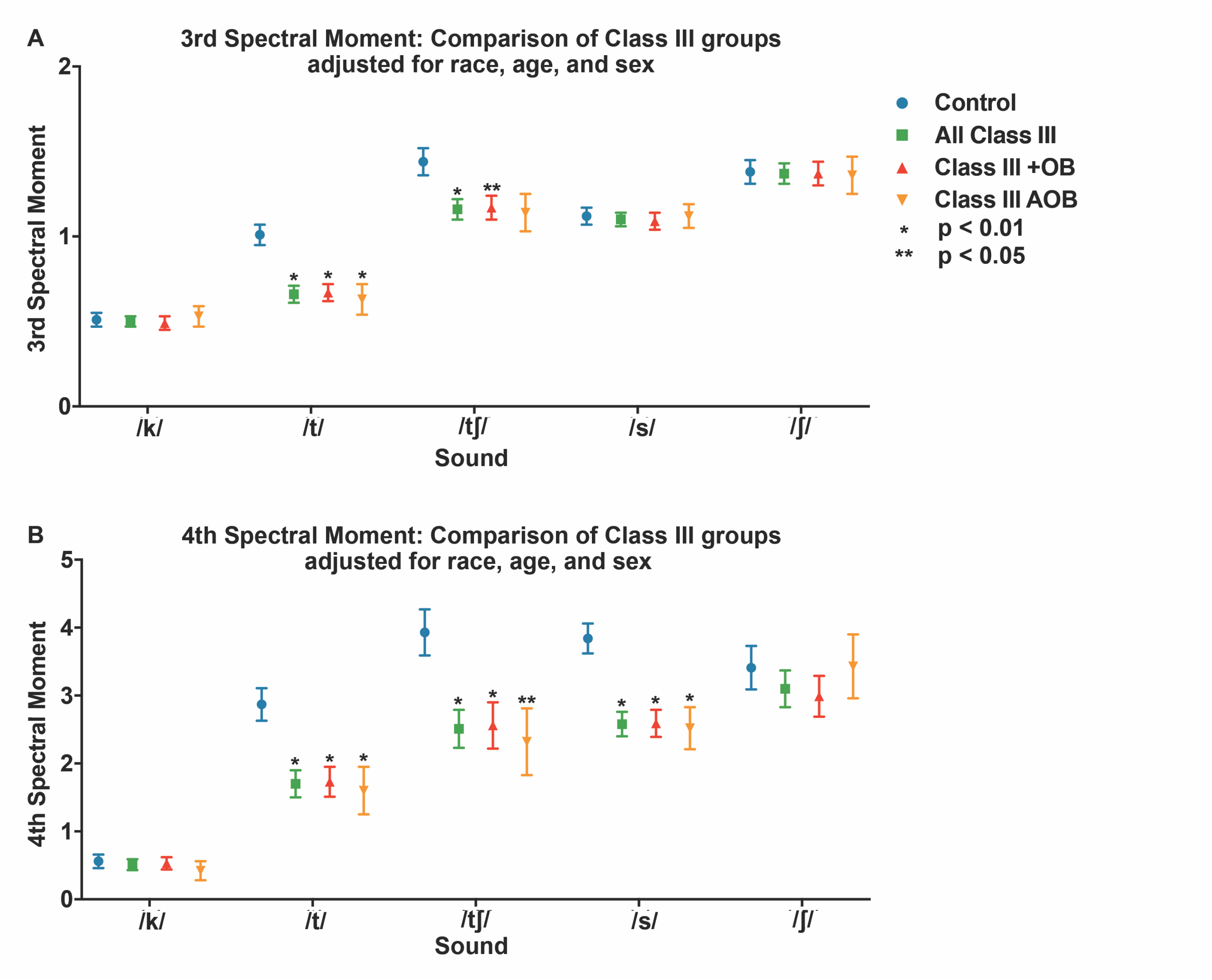

Supplemental Figure 2: 3rd and 4th Spectral Moments for patients with Class III DFD and control patients, adjusted for race, sex and age. [A.] 3rd spectral moment (M3, skew = symmetry or degree of distortion of curve) by phone. [B.] 4th spectral moment (M4, kurtosis = tails distribution, measure of outliers). Blue circle: Control. Green square: All Class III DFD patients. Red triangle: Class III DFD patients with positive (+) overbite. Orange upside down triangle: Class III DFD patients with an anterior open bite (AOB) or negative overbite. Bars represent standard error. Conventions: * p< 0.01 significant by Bonferonni adjustment. ** p<0.05. ^individual comparison has significance but no type III group significance (p>0.05). ^^ type III significance 0.01<p<0.05. Data are represented in Tables S4-S13.

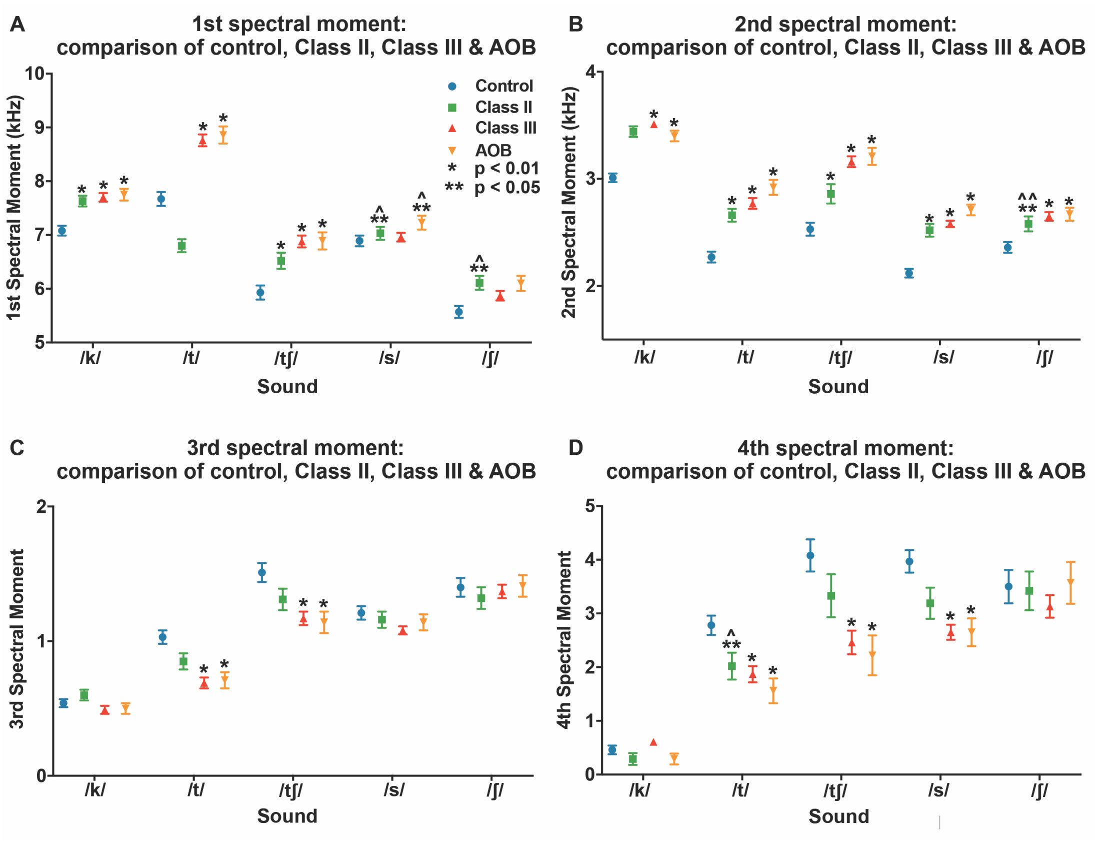

Supplemental Figure 3: 1st - 4th Spectral Moments for Patients with Class II, III and AOB DFD and Class I Control patients. [A.] 1st Spectral Moment / Mean Frequency (M1) by phone. [B.] 2nd spectral moment (M2, standard deviation = spectral spread) by phone. [C.] 3rd spectral moment (M3, skew = symmetry or degree of distortion of curve) by phone. [D.] 4th spectral moment (M4, kurtosis = tails distribution, measure of outliers). Blue circle: Control. Green square: All Class II DFD patients. Red triangle: All Class III DFD patients. Orange upside down triangle: All anterior open bite (AOB) patients. Bars represent standard error. Conventions: * p< 0.01 significant by Bonferonni adjustment. ** p<0.05. ^individual comparison has significance but no type III group significance (p>0.05). ^^ type III significance 0.01<p<0.05. Data are represented in Tables S4-S13 and in (29,30).

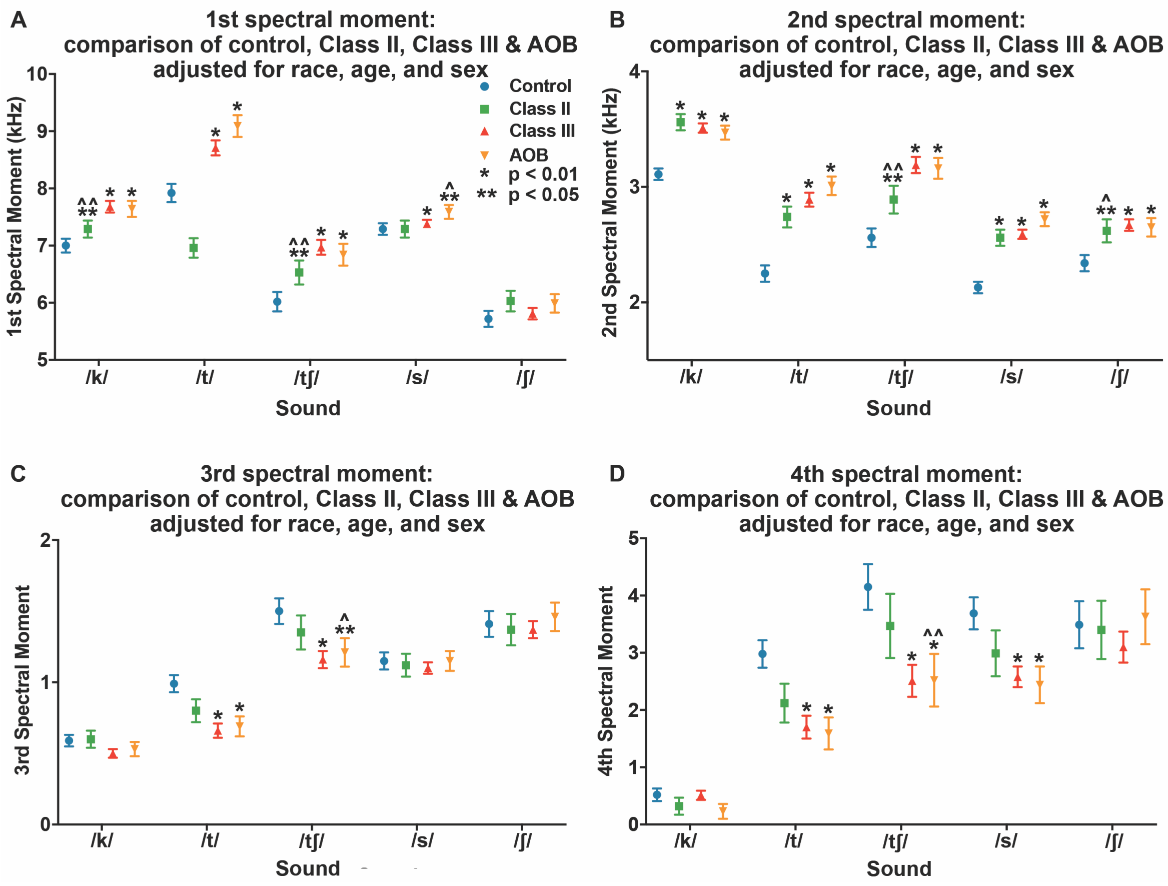

Supplemental Figure 4: 1st - 4th Spectral Moments for Patients with Class II, III and AOB DFD and Class I Control patients, adjusted for race, age and sex.[A.] 1st Spectral Moment / Mean Frequency (M1) by phone. [B.] 2nd spectral moment (M2, standard deviation = spectral spread) by phone. [C.] 3rd spectral moment (M3, skew = symmetry or degree of distortion of curve) by phone. [D.] 4th spectral moment (M4, kurtosis = tails distribution, measure of outliers). Blue circle: Control. Green square: All Class II DFD patients. Red triangle: All Class III DFD patients. Orange upside down triangle: All anterior open bite (AOB) patients. Bars represent standard error. Conventions: * p< 0.01 significant by Bonferonni adjustment. ** p<0.05. ^individual comparison has significance but no type III group significance (p>0.05). ^^ type III significance 0.01<p<0.05. Data are represented in Tables S4-S13 and in (29, 30).

Supplemental Table 1: Type and Prevalence of Distortions by Speech-Language Pathologist Perceptual Evaluation

Sound	Class III DFD Dental (n=102)	Control Dental (n=62)	Class III DFD Interdental (n=102)	Control Interdental (n=62)	Audio DFD (n=102)	Audio Control (n=62)
/sa/	63.73% (n=65)	1.61% (n=1)	3.92% (n=4)	0.00% (n=0)	66.67% (n=68)	1.61% (n=1)
/si/**	61.11% (n=11)	2.50% (n=1)	11.11% (n=2)	0.00% (n=0)	50.00% (n=9)	2.50% (n=1)
/sissy/**	55.56% (n=10)	2.50% (n=1)	11.11% (n=2)	0.00% (n=0)	50.00% (n=9)	2.50% (n=1)
/ta/	38.24% (n=39)	0.00% (n=0)	0.98% (n=1)	0.00% (n=0)	36.27% (n=37)	0.00% (n=0)
/ti/**	27.78% (n=5)	2.50% (n=1))	0.00% (n=0)	0.00% (n=0)	11.11% (n=2)	2.50% (n=1)
/la/	35.29% (n=36)	3.22% (n=2)	13.73% (n=14)	0.00% (n=0)	37.25% (n=38)	3.22% (n=2)

**For /si/, /sissy/, and /ti/, n=18 for Class III subjects and n=40 for controls. These sound were added to the evaluation partway through the study so we do not have data on all Class III and control subjects for these sounds.

Supplemental Table 2: Word List for Speech Evaluations

Word	Vowel	Sound
Chap	/a/	/Ch/
Cheap	/i/	/Ch/
Chew	/u/	/Ch/
Chop	/a/	/Ch/
Cap	/ae/	/K/
Coo	/u/	/K/
Cop	/a/	/K/
Key	/i/	/K/
Sack	/ae/	/S/
See	/i/	/S/
Sock	/a/	/S/
Sue	/u/	/S/
Shack	/ae/	/Sh/
She	/i/	/Sh/
Shock	/a/	/Sh/
Shoe	/u/	/Sh/
Tap	/ae/	/T/
Tea	/i/	/T/
Too	/u/	/T/
Top	/a/	/T/

Supplemental Table 3: Intra-examiner Cephalometric Concordance Correlations

Class III	ANB	IMPA	Wits	FMA	SN-GoGn	U1-SN (degrees)	U1-NA (degrees)	U1-NA (mm)
Lower CL	0.973	0.934	0.971	0.961	0.959	0.912	0.926	0.955
ConcCorr	0.989	0.971	0.987	0.983	0.982	0.962	0.968	0.981
Upper CL	0.995	0.988	0.994	0.993	0.992	0.983	0.986	0.992

Supplemental Table 4: Unadjusted and Adjusted Data for Class III DFD and Controls for phoneme /k/

Group	Variable	Unadjusted Mean (SE)	Unadjusted p-value^^	Adjusted Mean (SE)^	Adjusted p-value^,^^
Control N=62 (2448 Words)	M1 Mean (kHz)<br>M2 SD (kHz)<br>M3 Skew<br>M4 Kurtosis	7.27 (0.10)<br>3.27 (0.04)<br>0.49 (0.03)<br>0.64 (0.08)	Ref<br>Ref<br>Ref<br>Ref	7.22 (0.12)<br>3.24 (0.05)<br>0.51 (0.04)<br>0.56 (0.10)	Ref<br>Ref<br>Ref<br>Ref
Class III N=102 (1488 Words)	M1 Mean (kHz)<br>M2 SD (kHz)<br>M3 Skew<br>M4 Kurtosis	7.70 (0.08)<br>3.51 (0.03)<br>0.49 (0.03)<br>0.61 (0.06)	0.0006*<br><0.0001*<br>0.86<br>0.71	7.68 (0.10)<br>3.51 (0.04)<br>0.50 (0.03)<br>0.51 (0.08)	0.0005*<br><0.0001*<br>0.78<br>0.62

*Significant by Bonferroni adjustment (p<0.01)
**Significant by convention (0.01<p<0.05)
^Adjusted by Race, Sex and Age
^^Pairwise comparison with a Tukey-Kramer adjustment p-values were calculated using a mixed model comparing the DFD group with controls as reference, adjusting for age, race, and gender in adjusted groups. Controls have no p-values because they are the reference group for all other adjusted calculations.
N = number of patients in each group

Supplemental Table 5: Unadjusted and Adjusted Data for Class III DFD Subgroups and Controls for /k/

Group	Variable	Unadjusted Mean (SE)	Unadjusted Type III p-value^^	Unadjusted Pairwise p-value^^	Adjusted Mean (SE)^	Adjusted Type III p-value^,^^	Adjusted Pairwise p-value^,^^
Control N=62 (1488 Words)	M1 Mean (kHz)	7.27 (0.10)	0.003*	Ref	7.22 (0.12)	0.002*	Ref
	M2 SD (kHz)	3.25 (0.04)	<.0001*	Ref	3.21 (0.05)	<0.0001*	Ref
	M3 Skew	0.49 (0.03)	0.96	Ref	0.51 (0.04)	0.81	Ref
	M4 Kurtosis	0.64 (0.08)	0.85	Ref	0.56 (0.10)	0.68	Ref
Class III AOB N=22 (528 Words)	M1 Mean (kHz)	7.73 (0.17)		0.04**	7.65 (0.18)		0.08
	M2 SD (kHz)	3.57 (0.07)		0.0005*	3.60 (0.07)		<0.0001*
	M3 Skew	0.50 (0.06)		1.00	0.53 (0.06)		0.96
	M4 Kurtosis	0.56 (0.13)		0.84	0.42 (0.14)		0.65
Class III No AOB N=80 (1920 Words)	M1 Mean (kHz)	7.69 (0.09)		0.004*	7.70 (0.11)		0.002*
	M2 SD (kHz)	3.47 (0.04)		0.0005*	3.45 (0.05)		0.0001*
	M3 Skew	0.48 (0.03)		0.97	0.49 (0.04)		0.90
	M4 Kurtosis	0.62 (0.07)		0.97	0.53 (0.09)		0.96

*Significant by Bonferroni adjustment (p<0.01)
**Significant by convention (0.01<p<0.05)
^Adjusted by Race, Sex and Age
^^Pairwise with a Tukey-Kramer adjustment and Type III p-values were calculated using a mixed model comparing the DFD group with controls as reference, adjusting for age, race, and gender in adjusted groups. The Type III p-value indicates a significant difference overall between groups. Controls have no p-values because they are the reference group for all other adjusted calculations.
N = number of patients in each group

Supplemental Table 6: Unadjusted and Adjusted Data for Class III DFD and Controls for /t/

Group	Variable	Unadjusted Mean (SE)	Unadjusted p-value^^	Adjusted Mean (SE)^	Adjusted p-value^,^^
Control N=62 (744 Words)	M1 Mean (kHz)<br>M2 SD (kHz)<br>M3 Skew<br>M4 Kurtosis	7.56 (0.14)<br>2.18 (0.06)<br>1.10 (0.05)<br>3.05 (0.20)	Ref<br>Ref<br>Ref<br>Ref	7.71 (0.16)<br>2.28 (0.07)<br>1.01 (0.06)<br>2.87 (0.24)	Ref<br>Ref<br>Ref<br>Ref
Class III N=102 (1224 Words)	M1 Mean (kHz)<br>M2 SD (kHz)<br>M3 Skew<br>M4 Kurtosis	8.76 (0.11)<br>2.77 (0.05)<br>0.69 (0.04)<br>1.87 (0.15)	<0.0001*<br><0.0001*<br><0.0001*<br><0.0001*	8.71 (0.13)<br>2.89 (0.06)<br>0.66 (0.05)<br>1.70 (0.20)	<0.0001*<br><0.0001*<br><0.0001*<br><0.0001*

*Significant by Bonferroni adjustment (p<0.01)
**Significant by convention (0.01<p<0.05)
^Adjusted by Race, Sex and Age
^^Pairwise comparison with a Tukey-Kramer adjustment p-values were calculated using a mixed model comparing the DFD group with controls as reference, adjusting for age, race, and gender in adjusted groups. Controls have no p-values because they are the reference group for all other adjusted calculations.
N = number of patients in each group

Supplemental Table 7: Unadjusted and Adjusted Data for Class III DFD Subgroups and Controls for /t/

Group	Variable	Unadjusted Mean (SE)	Unadjusted Type III p-value^^	Unadjusted Pairwise p-value^^	Adjusted Mean (SE)^	Adjusted Type III p-value^,^^	Adjusted Pairwise p-value^,^^
Control N=62 (744 Words)	M1 Mean (kHz)<br>M2 SD (kHz)<br>M3 Skew<br>M4 Kurtosis	7.58 (0.14)<br>2.19 (0.06)<br>1.10 (0.05)<br>3.05 (0.20)	<0.0001*<br><0.0001*<br><0.0001*<br><0.0001*	Ref<br>Ref<br>Ref<br>Ref	7.71 (0.16)<br>2.28 (0.07)<br>1.01 (0.06)<br>2.87 (0.24)	<0.0001*<br><0.0001*<br><0.0001*<br><0.0001*	Ref<br>Ref<br>Ref<br>Ref
Class III AOB N=22 (264 Words)	M1 Mean (kHz)<br>M2 SD (kHz)<br>M3 Skew<br>M4 Kurtosis	9.10 (0.24)<br>2.76 (0.10)<br>0.65 (0.08)<br>1.76 (0.33)		<0.0001*<br><0.0001*<br><0.0001*<br>0.003*	8.93 (0.23)<br>2.90 (0.10)<br>0.63 (0.09)<br>1.60 (0.35)		<0.0001*<br><0.0001*<br>0.0005*<br>0.004*
Class III No AOB N=80 (960 Words)	M1 Mean (kHz)<br>M2 SD (kHz)<br>M3 Skew<br>M4 Kurtosis	8.69 (0.12)<br>2.77 (0.06)<br>0.70 (0.04)<br>1.90 (0.17)		<0.0001*<br><0.0001*<br><0.0001*<br><0.0001*	8.65 (0.15)<br>2.88 (0.07)<br>0.67 (0.05)<br>1.73 (0.22)		<0.0001*<br><0.0001*<br><0.0001*<br>0.0001*

*Significant by Bonferroni adjustment (p<0.01)
**Significant by convention (0.01<p<0.05)
^Adjusted by Race, Sex and Age
^^Pairwise with a Tukey-Kramer adjustment and Type III p-values were calculated using a mixed model comparing the DFD group with controls as reference, adjusting for age, race, and gender in adjusted groups. The Type III p-value indicates a significant difference overall between groups. Controls have no p-values because they are the reference group for all other adjusted calculations.
N = number of patients in each group

Supplemental Table 8: Unadjusted and Adjusted Data for Class III DFD and Controls for /ch/

Group	Variable	Unadjusted Mean (SE)	Unadjusted p-value^^	Adjusted Mean (SE)^	Adjusted p-value^,^^
Control N=62 (744 Words)	M1 Mean (kHz)<br>M2 SD (kHz)<br>M3 Skew<br>M4 Kurtosis	5.93 (0.14)<br>2.53 (0.07)<br>1.51 (0.07)<br>4.16 (0.28)	Ref<br>Ref<br>Ref<br>Ref	6.13 (0.16)<br>2.64 (0.08)<br>1.44 (0.08)<br>3.93 (0.34)	Ref<br>Ref<br>Ref<br>Ref
Class III N=102 (1224 Words)	M1 Mean (kHz)<br>M2 SD (kHz)<br>M3 Skew<br>M4 Kurtosis	6.88 (0.11)<br>3.16 (0.05)<br>1.17 (0.05)<br>2.46 (0.22)	<0.0001*<br><0.0001*<br><0.0001*<br><0.0001*	6.97 (0.13)<br>3.19 (0.07)<br>1.16 (0.06)<br>2.51 (0.28)	<0.0001*<br><0.0001*<br>0.002*<br>0.0002*

*Significant by Bonferroni adjustment (p<0.01)
**Significant by convention (0.01<p<0.05)
^Adjusted by Race, Sex and Age
^^Pairwise comparison with a Tukey-Kramer adjustment p-values were calculated using a mixed model comparing the DFD group with controls as reference, adjusting for age, race, and gender in adjusted groups. Controls have no p-values because they are the reference group for all other adjusted calculations.
N = number of patients in each group

<u>Supplemental Table 9:</u> Unadjusted and Adjusted Data for Class III DFD Subgroups and Controls for /ch/

Group	Variable	Unadjusted Mean (SE)	Unadjusted Type III p-value^^	Unadjusted Pairwise p-value^^	Adjusted Mean (SE)^	Adjusted Type III p-value^,^^	Adjusted Pairwise p-value^,^^
Control N=62 (744 Words)	M1 Mean (kHz)	5.93 (0.14)	<0.0001*	Ref	6.13 (0.16)	<0.0001*	Ref
	M2 SD (kHz)	2.53 (0.07)	<0.0001*	Ref	2.63 (0.08)	<0.0001*	Ref
	M3 Skew	1.51 (0.07)	0.0001*	Ref	1.44 (0.08)	0.007*	Ref
	M4 Kurtosis	4.16 (0.28)	<0.0001*	Ref	3.94 (0.34)	0.0007*	Ref
Class III AOB N=22 (264 Words)	M1 Mean (kHz)	6.98 (0.23)		0.0003*	6.94 (0.23)		0.007*
	M2 SD (kHz)	3.25 (0.11)		<0.0001*	3.28 (0.11)		<0.0001*
	M3 Skew	1.10 (0.11)		0.004*	1.14 (0.11)		0.060
	M4 Kurtosis	2.10 (0.47)		0.0006*	2.32 (0.49)		0.010*
Class III No AOB N=80 (960 Words)	M1 Mean (kHz)	6.86 (0.12)		<0.0001*	6.98 (0.15)		<0.0001*
	M2 SD (kHz)	3.14 (0.06)		<0.0001*	3.16 (0.07)		<0.0001*
	M3 Skew	1.19 (0.06)		0.0006*	1.17 (0.07)		0.010**
	M4 Kurtosis	2.55 (0.25)		<0.0001*	2.56 (0.34)		0.001*

*Significant by Bonferroni adjustment (p<0.01)
**Significant by convention (0.01<p<0.05)
^Adjusted by Race, Sex and Age
^^Pairwise with a Tukey-Kramer adjustment and Type III p-values were calculated using a mixed model comparing the DFD group with controls as reference, adjusting for age, race, and gender in adjusted groups. The Type III p-value indicates a significant difference overall between groups. Controls have no p-values because they are the reference group for all other adjusted calculations.
N = number of patients in each group

Supplemental Table 10: Unadjusted and Adjusted Data for Class III DFD and Controls for /s/

Group	Variable	Unadjusted Mean (SE)	Unadjusted p-value^^	Adjusted Mean (SE)^	Adjusted p-value^,^^
Control N=62 (1488 Words)	M1 Mean (kHz)<br>M2 SD (kHz)<br>M3 Skew<br>M4 Kurtosis	6.76 (0.10)<br>2.12 (0.04)<br>1.15 (0.04)<br>3.91 (0.18)	Ref<br>Ref<br>Ref<br>Ref	7.08 (0.09)<br>2.12 (0.05)<br>1.12 (0.05)<br>3.84 (0.22)	Ref<br>Ref<br>Ref<br>Ref
Class III N=102 (2448 Words)	M1 Mean (kHz)<br>M2 SD (kHz)<br>M3 Skew<br>M4 Kurtosis	6.96 (0.08)<br>2.58 (0.03)<br>1.08 (0.03)<br>2.65 (0.14)	0.10<br><0.0001*<br>0.19<br><0.0001*	7.38 (0.07)<br>2.59 (0.04)<br>1.10 (0.04)<br>2.58 (0.18)	0.003*<br><0.0001*<br>0.73<br><0.0001*

*Significant by Bonferroni adjustment (p<0.01)
**Significant by convention (0.01<p<0.05)
^Adjusted by Race, Sex and Age
^^Pairwise comparison with a Tukey-Kramer adjustment p-values were calculated using a mixed model comparing the DFD group with controls as reference, adjusting for age, race, and gender in adjusted groups. Controls have no p-values because they are the reference group for all other adjusted calculations.
N = number of patients in each group

Supplemental Table 11: Unadjusted and Adjusted Data for Class III DFD Subgroups and Controls for /s/

Group	Variable	Unadjusted Mean (SE)	Unadjusted Type III p-value^^	Unadjusted Pairwise p-value^^	Adjusted Mean (SE)^	Adjusted Type III p-value^,^^	Adjusted Pairwise p-value^,^^
Control N=62 (1488 Words)	M1 Mean (kHz)<br>M2 SD (kHz)<br>M3 Skew<br>M4 Kurtosis	6.75 (0.10)<br>2.12 (0.04)<br>1.15 (0.04)<br>3.91 (0.18)	0.24<br><0.0001*<br>0.42<br><0.0001*	Ref<br>Ref<br>Ref<br>Ref	7.12 (0.09)<br>2.12 (0.05)<br>1.11 (0.05)<br>3.85 (0.22)	0.003*<br><0.0001*<br>0.84<br><0.0001*	Ref<br>Ref<br>Ref<br>Ref
Class III AOB N=22 (528 Words)	M1 Mean (kHz)<br>M2 SD (kHz)<br>M3 Skew<br>M4 Kurtosis	7.01 (0.16)<br>2.67 (0.07)<br>1.08 (0.07)<br>2.54 (0.30)		0.33<br><0.0001*<br>0.69<br>0.0003*	7.28 (0.13)<br>2.67 (0.07)<br>1.12 (0.07)<br>2.52 (0.31)		0.51<br><0.0001*<br>0.99<br>0.0007*
Class III No AOB N=1920 (684 Words)	M1 Mean (kHz)<br>M2 SD (kHz)<br>M3 Skew<br>M4 Kurtosis	6.93 (0.09)<br>2.57 (0.04)<br>1.08 (0.04)<br>2.69 (0.16)		0.34<br><0.0001*<br>0.42<br><0.0001*	7.47 (0.08)<br>2.56 (0.05)<br>1.09 (0.05)<br>2.59 (0.20)		0.002*<br><0.0001*<br>0.89<br><0.0001*

*Significant by Bonferroni adjustment (p<0.01)
**Significant by convention (0.01<p<0.05)
^Adjusted by Race, Sex and Age
^^Pairwise with a Tukey-Kramer adjustment and Type III p-values were calculated using a mixed model comparing the DFD group with controls as reference, adjusting for age, race, and gender in adjusted groups. The Type III p-value indicates a significant difference overall between groups. Controls have no p-values because they are the reference group for all other adjusted calculations.
N = number of patients in each group

<u>Supplemental Table 12:</u> Unadjusted and Adjusted Data for Class III DFD and Controls for /sh/

Group	Variable	Unadjusted Mean (SE)	Unadjusted p-value^^	Adjusted Mean (SE)^	Adjusted p-value^,^^
Control N=62 (744 Words)	M1 Mean (kHz)<br>M2 SD (kHz)<br>M3 Skew<br>M4 Kurtosis	5.73 (0.11)<br>2.37 (0.05)<br>1.39 (0.06)<br>3.47 (0.27)	Ref<br>Ref<br>Ref<br>Ref	5.64 (0.13)<br>2.37 (0.06)<br>1.38 (0.07)<br>3.41 (0.32)	Ref<br>Ref<br>Ref<br>Ref
Class III N=102 (1224 Words)	M1 Mean (kHz)<br>M2 SD (kHz)<br>M3 Skew<br>M4 Kurtosis	5.87 (0.09)<br>2.65 (0.04)<br>1.37 (0.05)<br>3.13 (0.21)	0.36<br><0.0001*<br>0.85<br>0.32	5.81 (0.10)<br>2.67 (0.05)<br>1.37 (0.06)<br>3.10 (0.27)	0.21<br><0.0001*<br>0.91<br>0.38

*Significant by Bonferroni adjustment (p<0.01)
**Significant by convention (0.01<p<0.05)
^Adjusted by Race, Sex and Age
^^Pairwise comparison with a Tukey-Kramer adjustment p-values were calculated using a mixed model comparing the DFD group with controls as reference, adjusting for age, race, and gender in adjusted groups. Controls have no p-values because they are the reference group for all other adjusted calculations.
N = number of patients in each group

Supplemental Table 13: Unadjusted and Adjusted Data for Class III DFD Subgroups and Controls for /sh

Group	Variable	Unadjusted Mean (SE)	Unadjusted Type III p-value^^	Unadjusted Pairwise p-value^^	Adjusted Mean (SE)^	Adjusted Type III p-value^,^^	Adjusted Pairwise p-value^,^^
Control N=62 (744 Words)	M1 Mean (kHz)<br>M2 SD (kHz)<br>M3 Skew<br>M4 Kurtosis	5.74 (0.11)<br>2.37 (0.05)<br>1.39 (0.06)<br>3.47 (0.27)	0.26<br><0.0001*<br>0.93<br>0.50	Ref<br>Ref<br>Ref<br>Ref	5.63 (0.13)<br>2.37 (0.06)<br>1.38 (0.08)<br>3.40 (0.32)	0.34<br><0.0001*<br>0.99<br>0.47	Ref<br>Ref<br>Ref<br>Ref
Class III AOB N=22 (264 Words)	M1 Mean (kHz)<br>M2 SD (kHz)<br>M3 Skew<br>M4 Kurtosis	6.10 (0.19)<br>2.66 (0.08)<br>1.34 (0.11)<br>3.38 (0.45)		0.24<br>0.009*<br>0.92<br>0.98	5.93 (0.18)<br>2.68 (0.09)<br>1.36 (0.11)<br>3.43 (0.47)		0.33<br>0.007*<br>0.99<br>1.00
Class III No AOB N=80 (960 Words)	M1 Mean (kHz)<br>M2 SD (kHz)<br>M3 Skew<br>M4 Kurtosis	5.80 (0.10)<br>2.65 (0.04)<br>1.38 (0.06)<br>3.06 (0.24)		0.91<br>0.0001*<br>1.00<br>0.48	5.77 (0.12)<br>2.66 (0.06)<br>1.37 (0.07)<br>2.99 (0.30)		0.60<br>0.0002*<br>1.00<br>0.51

*Significant by Bonferroni adjustment (p<0.01)
**Significant by convention (0.01<p<0.05)
^Adjusted by Race, Sex and Age
^^Pairwise with a Tukey-Kramer adjustment and Type III p-values were calculated using a mixed model comparing the DFD group with controls as reference, adjusting for age, race, and gender in adjusted groups. The Type III p-value indicates a significant difference overall between groups. Controls have no p-values because they are the reference group for all other adjusted calculations.
N = number of patients in each group

Supplemental Table 14: Adjusted regression data for spectral moments as a function of ANB°*

Group	Variable	Y-intercept	Std Error	Beta	Std Error	p-value^
/k/	M1 Mean (kHz)	7.4422	0.3841	0.000069	.0141	0.9961
	M2 SD (kHz)	3.6593	0.1563	0.00274	0.005738	0.6331
	M3 Skew	0.5669	0.1291	0.004982	0.004738	0.2931
	M4 Kurtosis	0.4168	0.3033	-0.00065	0.01113	0.9534
/t/	M1 Mean	8.6465	0.483	-0.0373	0.01773	0.0356^^
	M2 SD	2.9115	0.2334	0.01809	0.008569	0.0349^^
	M3 Skew	0.5132	0.1639	0.003932	0.006018	0.5137
	M4 Kurtosis	1.3722	0.6211	-0.02727	0.0228	0.2318
/ch/	M1 Mean	6.7989	0.5101	-0.00049	0.01872	0.9791
	M2 SD	3.1171	0.2345	-0.00953	0.008609	0.2687
	M3 Skew	1.1263	0.229	0.008925	0.008405	0.2885
	M4 Kurtosis	3.0534	0.9092	0.0423	0.03338	0.2053
/s/	M1 Mean	7.0426	0.3168	0.01854	0.01163	0.1111
	M2 SD	2.481	0.1588	-0.00284	0.005831	0.6264
	M3 Skew	0.9921	0.1489	0.00323	0.005467	0.5546
	M4 Kurtosis	3.0625	0.6612	0.04633	0.02427	0.0564
/sh/	M1 Mean	6.2445	0.404	0.02799	0.01483	0.0594
	M2 SD	2.6831	0.1878	-0.0035	0.006895	0.6121
	M3 Skew	0.8679	0.2172	-0.00453	0.007972	0.5698
	M4 Kurtosis	1.5802	0.9466	0.02892	0.03475	0.4054

*Adjusted for age, race and sex. Model includes all patients with DFD (n= 169 total; 7 AOB Class I, 10 AOB Class II, 22 AOB Class III, 28 Class II +OB, 22 Class III +OB, 80 Class III +OB) and controls (n = 62).

^p-value indicates a linear relationship between the spectral moment and ANB°

^^p-value convention ($p<0.05$)

Supplemental Table 15: Adjusted regression data for spectral moments as a function of IMPA°*

Group	Variable	Y-intercept	Std Error	Beta	Std Error	p-value^
**/k/**	M1 Mean (kHz)	7.3288	0.7163	0.001406	0.007534	0.8519
	M2 SD (kHz)	3.726	0.2917	-0.00106	0.003068	0.7289
	M3 Skew	0.6177	0.2416	-0.00092	0.002541	0.716
	M4 Kurtosis	0.4457	0.5655	-0.00032	0.005948	0.9574
**/t/**	M1 Mean	9.0343	0.9128	-0.00278	0.0096	0.7721
	M2 SD	1.9806	0.4338	0.01044	0.004563	0.0223^^
	M3 Skew	0.5428	0.3062	-0.00058	0.00322	0.8587
	M4 Kurtosis	3.1067	1.1529	-0.01994	0.01213	0.1003
**/ch/**	M1 Mean	6.5823	0.951	0.002715	0.01	0.7861
	M2 SD	3.3068	0.439	-0.00179	0.004617	0.6981
	M3 Skew	1.1005	0.4287	-0.00021	0.004509	0.9625
	M4 Kurtosis	2.3159	1.7045	0.00657	0.01793	0.7141
**/s/**	M1 Mean	6.4496	0.5937	0.006416	0.006244	0.3043
	M2 SD	2.6459	0.296	-0.00185	0.003113	0.5524
	M3 Skew	0.9708	0.278	0.000064	0.002924	0.9824
	M4 Kurtosis	1.3519	1.2374	0.01843	0.01301	0.1568
**/sh/**	M1 Mean	5.4822	0.7604	0.00783	0.007997	0.3277
	M2 SD	3.0454	0.3489	-0.00426	0.003669	0.2461
	M3 Skew	1.0929	0.405	-0.00252	0.004259	0.5541
	M4 Kurtosis	-0.1792	1.7623	0.02009	0.01853	0.2787

*Adjusted for age, race and sex. Model includes all patients with DFD (n= 169 total; 7 AOB Class I, 10 AOB Class II, 22 AOB Class III, 28 Class II +OB, 22 Class III +OB, 80 Class III +OB) and controls (n = 62).

^p-value indicates a linear relationship between the spectral moment and IMPA°

^^p-value convention ($p<0.05$)

Supplemental Table 16: Adjusted regression data for spectral moments as a function of Wits (mm)*

Group	Variable	Y-intercept	Std Error	Beta	Std Error	p-value^
**/k/**	M1 Mean (kHz)	7.5017	0.3864	0.007473	0.009755	0.4437
	M2 SD (kHz)	3.56	0.1569	-0.00606	0.003962	0.1263
	M3 Skew	0.5454	0.1306	0.000233	0.003298	0.9438
	M4 Kurtosis	0.3906	0.3058	-0.00323	0.00772	0.6758
**/t/**	M1 Mean	8.6091	0.4879	-0.02539	0.01232	0.0395^^
	M2 SD	2.881	0.2376	0.006799	0.006	0.2574
	M3 Skew	0.5267	0.1649	0.004103	0.004163	0.3245
	M4 Kurtosis	1.3913	0.06274	-0.01333	0.1584	0.4002
**/ch/**	M1 Mean	6.7775	0.5139	-0.00271	0.01297	0.8345
	M2 SD	3.0045	0.01297	-0.01879	0.005786	0.0012^^
	M3 Skew	1.1265	0.231	0.004962	0.005833	0.3951
	M4 Kurtosis	3.2095	0.9103	0.04239	0.02298	0.0654
**/s/**	M1 Mean	7.0528	0.32	0.01131	0.008078	0.1616
	M2 SD	2.4209	0.1574	-0.0096	0.003973	0.0157^^
	M3 Skew	0.9893	0.1502	0.001529	0.003791	0.6868
	M4 Kurtosis	3.1299	0.6671	0.03422	0.01684	0.0423^^
**/sh/**	M1 Mean	6.2435	0.409	0.01536	0.01033	0.1372
	M2 SD	2.6364	0.1872	-0.00874	0.004727	0.0648
	M3 Skew	0.8388	0.2181	-0.00603	0.005508	0.2736
	M4 Kurtosis	1.5641	0.9549	0.01497	0.2411	0.5348

*Adjusted for age, race and sex. Model includes all patients with DFD (n= 169 total; 7 AOB Class I, 10 AOB Class II, 22 AOB Class III, 28 Class II +OB, 22 Class III +OB, 80 Class III +OB) and controls (n = 62).
^p-value indicates a linear relationship between the spectral moment and Wits (mm).
^^p-value convention ($p<0.05$)

Supplemental Table 17: Adjusted regression data for spectral moments as a function of FMA°*

Group	Variable	Y-intercept	Std Error	Beta	Std Error	p-value^
**/k/**	M1 Mean (kHz)	7.30	0.41	0.0083	0.0099	0.4008
	M2 SD (kHz)	3.50	0.1619	0.01	0.0039	0.0113^^
	M3 Skew	.4502	0.1356	0.0054	0.0033	0.0989
	M4 Kurtosis	0.4525	0.3211	-0.00211	0.00782	0.7872
**/t/**	M1 Mean	8.8242	0.5185	-0.00107	0.0126	0.9326
	M2 SD	2.562	0.2436	0.01745	0.005931	0.0033^^
	M3 Skew	0.5774	0.1731	-0.00517	0.004214	0.2204
	M4 Kurtosis	1.9857	0.6517	-0.032	0.01587	0.0439
**/ch/**	M1 Mean	6.507	0.5361	0.019	0.01305	0.1458
	M2 SD	3.0191	0.2473	0.00946	0.00602	0.1164
	M3 Skew	1.1109	0.2434	-0.00178	0.005925	0.7636
	M4 Kurtosis	3.1793	0.9654	-0.02224	0.0235	0.3441
**/s/**	M1 Mean	7.0701	0.3377	-0.00614	0.008223	0.4554
	M2 SD	2.3831	0.1673	0.00674	0.0041	0.0982
	M3 Skew	0.8519	0.1553	0.00829	0.00378	0.0285
	M4 Kurtosis	2.617	0.7044	0.0148	0.0172	0.3868
**/sh/**	M1 Mean	6.0941	0.4333	0.00147	0.0106	0.8895
	M2 SD	2.703	0.1991	-0.0003	0.00485	0.95
	M3 Skew	0.7967	0.2293	0.00616	0.00558	0.2698
	M4 Kurtosis	1.0904	1.0014	0.02426	0.0244	0.3199

*Adjusted for age, race and sex. Model includes all patients with DFD (n= 169 total; 7 AOB Class I, 10 AOB Class II, 22 AOB Class III, 28 Class II +OB, 22 Class III +OB, 80 Class III +OB) and controls (n = 62).
^p-value indicates a linear relationship between the spectral moment and FMA°
^^p-value convention ($p<0.05$)

<u>Supplemental Table 18:</u> Adjusted regression data for spectral moments as a function of SN-GoGn°*

Group	Variable	Y-intercept	Std Error	Beta	Std Error	p-value^
**/k/**	M1 Mean (kHz)	7.6613	0.4489	-0.00814	0.0093	0.3802
	M2 SD (kHz)	3.452	0.1809	0.0075	0.0037	0.0458^^
	M3 Skew	0.4067	0.1502	0.00513	0.0031	0.0986
	M4 Kurtosis	0.3485	0.3551	0.0028	0.0073	0.7059
**/t/**	M1 Mean	9.6586	0.5587	-0.03253	0.01155	0.0049^^
	M2 SD	2.7152	0.277	0.0041	0.00573	0.4696
	M3 Skew	0.3954	0.1917	0.00385	0.003964	0.3312
	M4 Kurtosis	1.368	0.731	0.0054	0.01511	0.7211
**/ch/**	M1 Mean	6.9866	0.5968	-0.00703	0.01234	0.5692
	M2 SD	3.0839	0.2757	0.00299	0.0057	0.6001
	M3 Skew	0.9248	0.2682	0.0060	0.005544	0.2762
	M4 Kurtosis	2.5351	1.0702	0.01193	0.02212	0.5898
**/s/**	M1 Mean	7.2193	0.3723	-0.00942	0.007696	0.221
	M2 SD	2.475	0.1863	0.0007	0.00385	0.8556
	M3 Skew	0.74	0.1707	0.00905	0.0035	0.0104
	M4 Kurtosis	2.0977	0.7725	0.02817	0.01597	0.0779
**/sh/**	M1 Mean	6.3542	0.478	-0.0089	0.0099	0.3656
	M2 SD	2.7963	0.2197	-0.00378	0.00454	0.4053
	M3 Skew	0.6323	0.2515	0.0099	0.0052	0.0583
	M4 Kurtosis	0.452	1.1004	0.039	0.0228	0.0853

*Adjusted for age, race and sex. Model includes all patients with DFD (n= 169 total; 7 AOB Class I, 10 AOB Class II, 22 AOB Class III, 28 Class II +OB, 22 Class III +OB, 80 Class III +OB) and controls (n = 62).
^p-value indicates a linear relationship between the spectral moment and SN-GoGn°*
^^p-value convention ($p<0.05$)

Supplemental Table 19: Adjusted regression data for spectral moments, mix models for OJ*

Group	Variable	Y-intercept	Std Error	Beta	Std Error	p-value^
**/k/**	M1 Mean (kHz)	7.0712	0.3423	-0.01487	0.01393	0.286
	M2 SD (kHz)	3.4361	0.1713	-0.01877	0.0070	0.0072^^
	M3 Skew	0.6157	0.1099	-0.00162	0.0045	0.7173
	M4 Kurtosis	0.3474	0.2667	-0.00939	0.0109	0.3872
**/t/**	M1 Mean	8.2978	0.4185	-0.0689	0.01703	<0.0001^^
	M2 SD	2.7876	0.2153	-0.01823	0.0088	0.0377^^
	M3 Skew	0.2575	0.1936	0.01365	0.0079	0.0834
	M4 Kurtosis	2.0805	0.6321	0.0279	0.02573	0.2793
**/ch/**	M1 Mean	6.5532	0.4139	-0.0361	0.01685	0.0326^^
	M2 SD	3.1352	0.2101	-0.03949	0.0086	<0.0001^^
	M3 Skew	1.139	0.2006	0.01135	0.0082	0.1645
	M4 Kurtosis	2.7086	0.8219	0.0655	0.0335	0.0504
**/s/**	M1 Mean	6.3941	0.2875	0.0034	0.0117	0.7714
	M2 SD	2.5126	0.1532	-0.02387	0.0062	0.0001^^
	M3 Skew	0.9193	0.1438	-0.00394	0.00585	0.5014
	M4 Kurtosis	2.3331	0.5697	0.05498	0.02319	0.0178^
**/sh/**	M1 Mean	5.7328	0.3241	0.01022	0.01319	0.4385
	M2 SD	2.5668	0.1679	-0.01927	0.006835	0.0049^^
	M3 Skew	1.0787	0.1845	-0.00833	0.00751	0.2675
	M4 Kurtosis	2.0812	0.7834	0.02471	0.03189	0.4385

*Adjusted for age, race and sex. Model includes all patients with DFD (n= 169 total; 7 AOB Class I, 10 AOB Class II, 22 AOB Class III, 28 Class II +OB, 22 Class III +OB, 80 Class III +OB) and controls (n = 62).
^p-value indicates a linear relationship between the spectral moment and OJ (mm).
^^p-value convention (p<0.05)
